# 8,000 years of climate, vegetation, fire and land-use dynamics in the thermo-mediterranean vegetation belt of northern Sardinia (Italy)

**DOI:** 10.1007/s00334-021-00832-3

**Published:** 2021-04-21

**Authors:** Tiziana Pedrotta, Erika Gobet, Christoph Schwörer, Giorgia Beffa, Christoph Butz, Paul D. Henne, César Morales-Molino, Salvatore Pasta, Jacqueline F. N. van Leeuwen, Hendrik Vogel, Elias Zwimpfer, Flavio S. Anselmetti, Martin Grosjean, Willy Tinner

**Affiliations:** 1 Institute of Plant Sciences and Oeschger Centre for Climate Change Research, University of Bern, Altenbergrain 21, 3013 Bern, Switzerland; 2 Institute of Geography and Oeschger Centre for Climate Change Research, University of Bern, Hallerstraße 12, 3012 Bern, Switzerland; 3 U.S. Geological Survey, Geosciences and Environmental Change Science Center, Denver Federal Center, MS 980, P.O. Box 25046, Denver, CO 80225 USA; 4 Institute of Biosciences and BioResources (IBBR), Division of Palermo, National Research Council (CNR), Corso Calatafimi, 414, 90129 Palermo (PA), Italy; 5 Institute of Geological Sciences and Oeschger Centre for Climate Change Research, University of Bern, Baltzerstraße 1+3, 3012 Bern, Switzerland

**Keywords:** Palaeoecology, Palaeolimnology, Drought, Browsing, Cultivation, Fire, Erosion, Island ecology

## Abstract

Knowledge about the vegetation history of Sardinia, the second largest island of the Mediterranean, is scanty. Here, we present a new sedimentary record covering the past ~ 8,000 years from Lago di Baratz, north-west Sardinia. Vegetation and fire history are reconstructed by pollen, spores, macrofossils and charcoal analyses and environmental dynamics by high-resolution element geochemistry together with pigment analyses. During the period 8,100–7,500 cal bp, when seasonality was high and fire and erosion were frequent, *Erica arborea* and *E. scoparia* woodlands dominated the coastal landscape. Subsequently, between 7,500 and 5,500 cal bp, seasonality gradually declined and thermo-mediterranean woodlands with *Pistacia* and *Quercus ilex* partially replaced *Erica* communities under diminished incidence of fire. After 5,500 cal bp, evergreen oak forests expanded markedly, erosion declined and lake levels increased, likely in response to increasing (summer) moisture availability. Increased anthropogenic fire disturbance triggered shrubland expansions (e.g. *Tamarix* and *Pistacia*) around 5,000–4,500 cal bp. Subsequently around 4,000–3,500 cal bp evergreen oak-olive forests expanded massively when fire activity declined and lake productivity and anoxia reached Holocene maxima. Land-use activities during the past 4,000 years (since the Bronze Age) gradually disrupted coastal forests, but relict stands persisted under rather stable environmental conditions until ca. 200 cal bp, when agricultural activities intensified and *Pinus* and *Eucalyptus* were planted to stabilize the sand dunes. Pervasive prehistoric land-use activities since at least the Bronze Age Nuraghi period included the cultivation of *Prunus*, *Olea europaea* and *Juglans regia* after 3,500–3,300 cal bp, and *Quercus suber* after 2,500 cal bp. We conclude that restoring less flammable native *Q. ilex* and *O. europaea* forest communities would markedly reduce fire risk and erodibility compared to recent forest plantations with flammable non-native trees (e.g. *Pinus*, *Eucalyptus*) and xerophytic shrubland (e.g. *Cistus*, *Erica*).

## Introduction

The study site, Lago di Baratz, is located in the thermo-mediterranean vegetation belt of Sardinia, which is characterized by evergreen species of the maquis (tall shrublands) and garrigue (low shrublands) that are well adapted to hot, dry summers and mild, humid winters (Lang 1994). Vegetation ecologists assume that before pervasive land use, evergreen broadleaved forests (e.g. *Quercus ilex*, *Olea europaea*) were prevalent (e.g. Chiappini 1985; Bacchetta et al. 2009). This assumption mainly derives from the observation that relict forest stands are able to grow in the thermo-mediterranean belt under environmental conditions that are characteristic of maquis and garrigue communities. Knowledge about natural conditions and the resilience of these relict evergreen broadleaved, thermo-mediterranean forests is particularly scarce, due to intensive land use and deforestation over the past millennia. Growing in the warmest and driest environments of Europe, this vegetation type is considered very vulnerable to climate-change impacts, mainly because of aridity and disturbance-driven forest decline and biodiversity loss (Schröter et al. 2005; Henne et al. 2015; Baudena et al. 2020). Direct human impacts compound climatic threats to these relict forests, for instance through arson or the introduction of alien trees (e.g. *Eucalyptus*, *Pinus*) that enhance forest flammability (Fernandes 2009; Moreira et al. 2009).

Palaeoecological studies are in agreement with ecological assessments and emphasize that in the Mediterranean region, as elsewhere in Europe, woodlands were strongly altered or completely removed during the past 5,000 years as a consequence of anthropogenic land use (Birks and Tinner 2016). Studies at several sites in Sicily and on the Italian mainland, including the use of dynamic vegetation models, support the ecological hypothesis that today’s open environments resulted primarily from millennia of land use (e.g. Colombaroli et al. 2007; Tinner et al. 2009, 2013, 2016; Bisculm et al. 2012; Calò et al. 2012; Henne et al. 2015; Samartin et al. 2017). Increasing aridity (Jalut et al. 2009; Mercuri et al. 2012; Jiménez-Moreno et al. 2015; Ramos-Román et al. 2018; Schröder et al. 2018) or a combination of both climatic change and land use (e.g. Carrión et al. 2010) may also have affected Mediterranean ecosystems. Recent multiproxy studies that combine vegetation with palaeo-environmental reconstructions provide valuable evidence to disentangle causes and consequences of ecosystem dynamics (Zanchetta et al 2012; Aranbarri et al. 2014; Jouffroy-Bapicot et al. 2016; Melis et al. 2017; Roberts et al. 2019). Of particular importance for Mediterranean vegetation composition are moisture conditions that can be reconstructed by former lake properties such as salinity or water depth (e.g. Magny et al. 2007a, b, 2012; Curry et al. 2016). Reconstruction of erosion dynamics may reveal the effects of afforestation or deforestation activities (e.g. Roberts et al. 2019), while the study of past lake productivity may deliver important information about eutrophication, for example as a result of human impact (e.g. Gassner et al. 2020).

Palaeoecological records show that all Mediterranean islands (e.g. Corsica, the Balearic Islands, Malta and even islets such as Cavallo between Corsica and Sardinia) had distinct vegetation dynamics during the Holocene (Reille et al. 1997, 1999; Djamali et al. 2013; Gambin et al. 2016; Burjachs et al. 2017; Poher et al. 2017; Servera-Vives et al. 2018; Revelles et al. 2019). An inherent value of islands is that they offer the possibility of addressing past vegetation dynamics with reduced or absent migration lags for species that locally survived the Quaternary glacial-interglacial cycles (Birks and Tinner 2016). For instance, recently published studies from Sardinia (e.g. Di Rita and Melis 2013; Beffa et al. 2016; Melis et al. 2018; Kalis and Schoch 2019) document an early *Erica arborea* and *E. scoparia* dominance that was gradually replaced by evergreen oak (*Quercus ilex*) forests during the Mid and Late Holocene (for chronology of Holocene periods see Walker et al. 2018)*.* Similar vegetation dynamics also occurred in Corsica but are lacking in neighbouring Italy, Sicily, Malta or the Balearic Islands (e.g. Kelly and Huntley 1991; Reille et al. 1997, 1999; Magri 1999; Magri and Sadori 1999; Allen et al. 2002; Drescher-Schneider et al. 2007; Noti et al. 2009; Calò et al. 2012; Djamali et al. 2013; Sadori et al. 2013; Gambin et al. 2016; Burjachs et al. 2017; Poher et al. 2017; Servera-Vives et al. 2018; Revelles et al. 2019). The expansion of *Erica* woodlands or maquis under very warm and dry conditions in the thermo-mediterranean vegetation belt at the transition from the Early to the Mid Holocene may have resulted from extreme drought and high fire incidence (Beffa et al. 2016). Assessing the causes of establishment and decline of this vegetation type is of paramount importance. For instance, recurrent and drastic fire and drought disturbance may re-establish under global warming conditions, meaning high hazards for ecosystems and high risks for economy and society (Henne et al. 2015; Beffa et al. 2016).

The main goals of our study are: (1) to provide an account of changes in detrital input, productivity, hypolimnetic anoxia (lake stratification), lake levels and salinity using high-resolution bio-geochemical proxies; (2) to provide the first continuous ca. 8,000 year long record of the vegetation and fire history of western coastal Sardinia on the basis of pollen, spores, charcoal and macrofossils; (3) to disentangle climatic, environmental and human impacts on vegetation and the fire regime with a special focus on major vegetation changes (e.g. decline of *Erica* woodlands, expansion of *Quercus ilex—Olea* forests); (4) to compare our new results with other studies in Sardinia; and (5) to briefly compare the Sardinian vegetation history with the vegetation dynamics of other neighbouring Mediterranean islands (e.g. Corsica, Sicily), and identify drivers of vegetation changes in the wider thermo-mediterranean vegetation belt.

## Study site

**Fig. 1:**
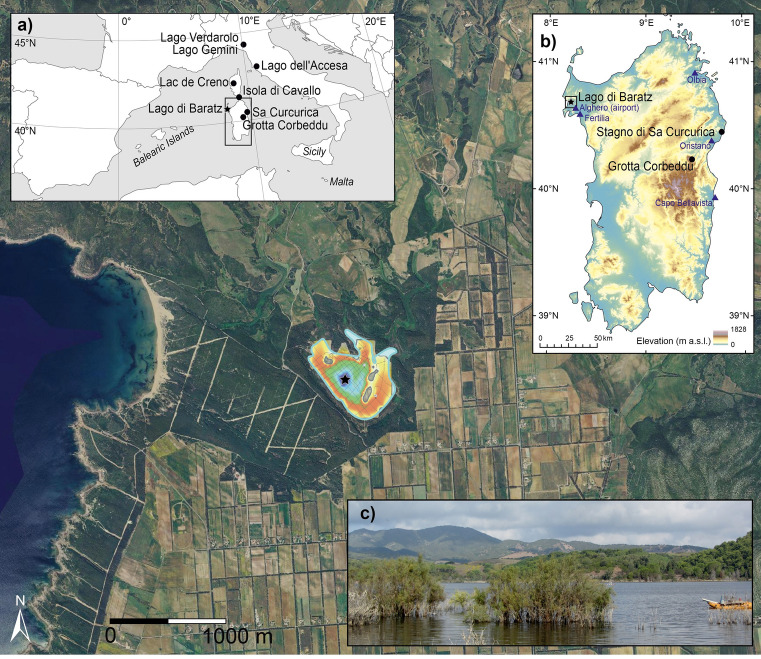
Map of the study area with Lago di Baratz (*black star*; orthophotograph from the Terraitaly (2006), modified), including **a** overview of important western Mediterranean palaeoecological sites mentioned in the text, **b** detailed location of the study site and meteorological stations in Sardinia (*blue triangles*) and **c** view from the shore of Lago di Baratz

Lago di Baratz is situated in north-western Sardinia (province of Nurra) at 27 m a.s.l., ca. 1 km from the sea. The lake had a surface of ca. 0.6 km^2^ and a water depth of 8.2 m in October 2012 (Fig. 1). It is the largest natural lake in Sardinia and under protection as part of the Natura2000 network (Site of Community Importance ITB 011155 “Lago di Baratz-Porto Ferro”). The lake probably originated when coastal sand dunes blocked the two river valleys of Rio dei Giunchi and Rio Puddighinu. The landscape is rather flat, with Monte de su Abba (86 m a.s.l.) north-east and sand dunes south-west of the lake (APM 2020). Pleistocene conglomerates, sand and mud deposits in river terraces prevail south and east of the lake (De Rosa and Cultrone 2014). West and north of the lake quartz-rich upper Permian-lower Triassic sandstones (“Buntsandstein”) occur that are partly covered by Holocene aeolian deposits (Niedda and Pirastru 2013). Jurassic marls and limestones form hills ca. 5 km east of the lake (De Rosa and Cultrone 2014). The lake waters are currently eutrophic and brackish, and show elevated values of nitrate and phosphate, primarily due to agricultural fertilizers (Giadrossich et al. 2015). Lago di Baratz is endorheic with significant oscillations of its level, highlighted by a near-desiccation event in 2008. However, the maximum water depth can reach 16 m with a water level of 33 m a.s.l., above which the water overflows towards the sea (Niedda and Pirastru 2013).

Sardinia has a typical Mediterranean climate, with mild and rainy winters and warm and dry summers with evaporation exceeding precipitation from April to September (Niedda and Pirastru 2013). Mean annual precipitation rises with increasing altitude and is lowest at the coast (SardegnaARPA 2020). In Alghero (40 m a.s.l., ca. 7 km SE from Lago di Baratz) mean annual rainfall (1981–2010) is 506 ± 173 mm, whereas mean January temperature is 9.4 ± 1.2 °C, mean July temperature 24.2 ± 1.2 °C, and mean annual temperature 16.1 ± 0.5 °C (SCIA 2020, Table 1). Species typical of the thermo-mediterranean vegetation belt growing around Lago di Baratz include *Quercus ilex*, *Olea europaea* var. *sylvestris*, *Chamaerops humilis*, *Myrtus communis*, *Phillyrea latifolia*, *Pistacia lentiscus*, *Juniperus oxycedrus* ssp. *macrocarpa* and *J*. *phoenicea*. Vegetation near Lago di Baratz includes recent *Pinus pinea* plantations on dunes. Alien plants such as *Acacia saligna*, *Eucalyptus camaldulensis*, *Myoporum* sp., *Carpobrotus* cf. *edulis* and *Ailanthus altissima* were introduced during the last century. Agricultural fields and grasslands occupy much of the flat area south and east of the lake (Fig. 1).

**Table 1:** Climate data from meteorological stations close to Lago di Baratz (western Sardinia) and Sa Curcurica (eastern Sardinia), for the reference period 1981–2010, given with ± 1 standard deviation (SCIA 2020)

Station	Alghero (W)	Fertilia (W)	Capo Bellavista (E)	Orosei (E)	Olbia (E)
Elevation (m a.s.l.)	40	39	150	19	13
Mean annual temperature (°C)	16.1 ± 0.5	–	17.5 ± 0.7	–	16.5 ± 0.6
Mean July temperature (°C)	24.2 ± 1.2	–	25.7 ± 1.2	–	25.3 ± 1.3
Mean January temperature (°C)	9.4 ± 1.2	–	10.7 ± 1	–	9.5 ± 1.2
Total annual precipitation (mm)	506 ± 173	553 ± 128	416 ± 136	553 ± 201	—
Total winter (DJF) precipitation (mm)	166 ± 81	167 ± 57	127 ± 69	181 ± 96	—
Total summer (JJA) precipitation (mm)	34 ± 28	33 ± 35	30 ± 30	35 ± 32	—

## Methods

### Coring, lithology and chronology

**Fig. 2:**
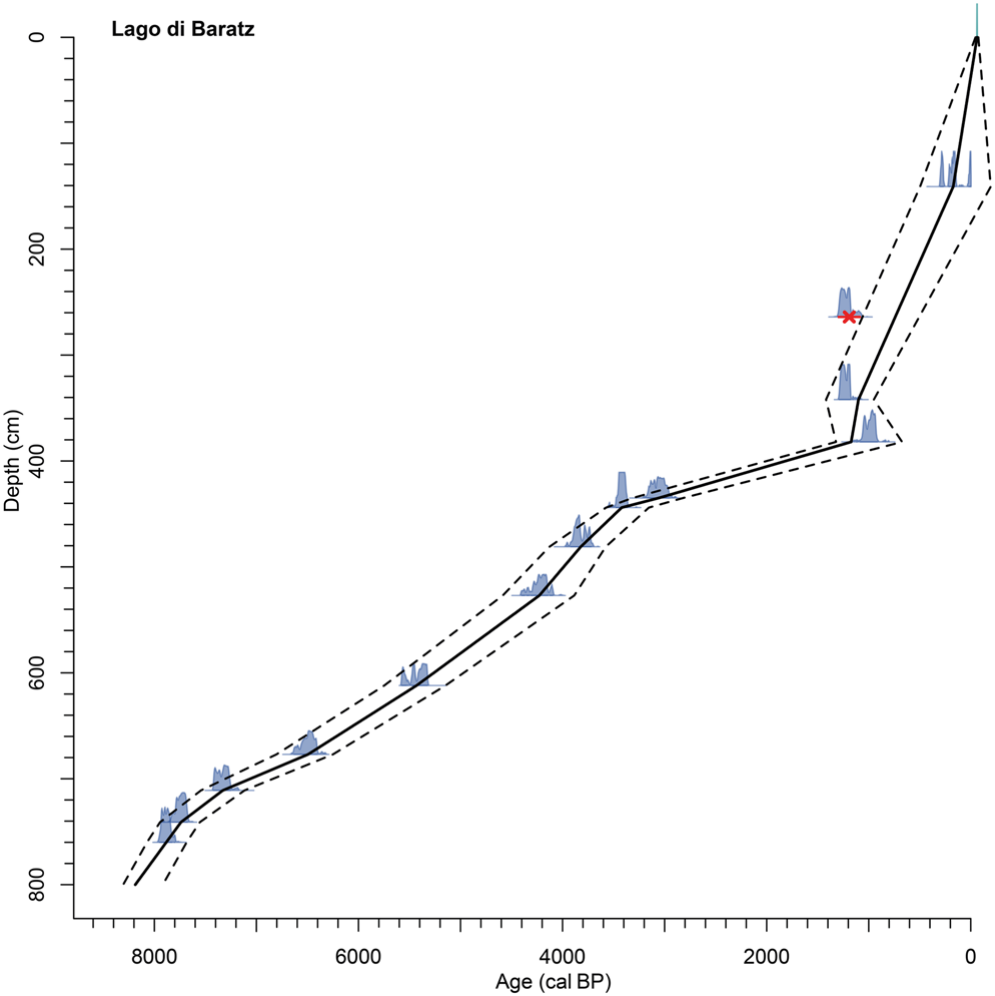
Depth-age model for core BRZ-D of Lago di Baratz (Italy) calculated with Clam 2.2 (Blaauw 2010) based on 13 radiocarbon dates from terrestrial macrofossil samples. The model takes into account the 2σ-confidence range of the calibrated ages (blue probability distributions). *Dashed lines* indicate the 95% confidence envelope of the generalized mixed effect regression (GAM, Heegaard et al. 2005)

In October 2012, two parallel cores (BRZ-D and BRZ-E) were recovered from the deepest part of the lake (water depth 8.2 m, 40°40′49.8″N, 008°13′31.1″E; Fig. 1) with a modified Streif-Livingstone piston corer (Lang 1994). Surface cores were taken with a Kajak corer to recover the sediment–water interface. The coring stopped in a gravel layer. The two parallel cores were visually correlated based on lithological features, providing a total composite sediment depth of 802 cm (Table 2). Thirteen accelerator mass spectrometry (AMS) ^14^C dates were measured at the Poznań Radiocarbon Laboratory (Poz) and at the Laboratory for the Analysis of Radiocarbon with AMS at the University of Bern (LARA, Table 3). Dates were calibrated using the INTCAL13 calibration curve (Reimer et al. 2013) and included in a generalized mixed-effect regression (GAM, Heegaard et al. 2005), which considers both the sampling depth of the dated material and the 2σ-confidence range of calibrated ages. The date at 262–266 cm (1,290 ± 50 cal bp) on a charcoal fragment was considered as too old after depth-age modeling and is therefore excluded. Due to its potentially long residence time in soils and in-built ages deriving from wood of old trees, charcoal may produce ages older than short-lived terrestrial macrofossil remains (Gavin 2001; Oswald et al. 2005). We linearly interpolated through the weighted average of the density distribution of the calibration ranges except for the dates at 380–384 and 338–346 cm, where the linear interpolation goes through the outermost limits of the probability distributions at 1,172 and 1,100 cal bp to avoid a temporal inversion (Fig. 2). Below the oldest radiocarbon date (7,797–7,952 cal bp, Table 3), the ages were linearly extrapolated, resulting in a maximum age of ca. 8,200 cal bp (Fig. 2). After building the chronology and applying it to the sedimentary records, the new calibration curve INTCAL20 was released (Reimer et al. 2020). The recalibrated radiocarbon dates show very small differences that do not affect the age-depth model (Table 3).

**Table 2:** Sediment description

Depth (cm)	Age (cal bp)	Lithology
0–485	−62–3,850	Silty gyttja
485–543	3,850–4,450	Clayey/silty gyttja
543–549	4,450–4,550	Clayey/silty gyttja with shells
549–553	4,550–4,600	Silty gyttja
553–583	4,600–5,000	Silty gyttja with shells
583–641	5,000–5,900	Dark silty gyttja
641–708	5,900–7,250	Silty/clayey gyttja
708–708.5	7,250	Vivianite
708.5–746	7,250–7,800	Silty gyttja
746–802	7,800–8,200	Gravel

**Table 3:** Radiocarbon dates and calibrated ages in diagram

Lab. code	Depth (cm)	Material	^14^C age (yrs bp)	Cal. age, 2σ (cal yrs bp)^a^	Cal. age, 2σ (cal yrs bp)^b^	Age in diagram according to clam (cal yrs bp)
Poz-63867	138–144	El, Bs, W, C	200 ± 30	−4–302	0–305	172
Poz-63868*	262–266	C	1,290 ± 50	1,083–1,298	1,075–1,298	–
Poz-63870	338–346	El, Bs, P, C	1,285 ± 35	1,100–1,294	1,129–1,290	1,100
BE2749.1.1	380–384	Lf, W	1,070 ± 50	835–1,172	828–1,174	1,172
BE3004.1.1	432–438	El, Bl	2,915 ± 35	2,948–3,176	2,939–3,205	3,060
BE3005.1.1	442–446	El, Et, P	3,200 ± 35	3,360–3,544	3,364–3,478	3,421
Poz-59762	480–482	El	3,545 ± 35	3,716–3,956	3,701–3,962	3,822
Poz-63871	526–528	El, C	3,820 ± 40	4,092–4,405	4,091–4,403	4,228
Poz-63872	610–614	El, Bs, W, P, C	4,700 ± 40	5,320–5,580	5,320–5,574	5,431
BE2750.1.1	674–680	W, P, C	5,700 ± 50	6,399–6,637	6,354–6,634	6,495
Poz-56283	710–712	Bs	6,390 ± 50	7,249–7,425	7,174–7,423	7,329
Poz-56284	740–742	W, T	6,910 ± 40	7,672–7,831	7,669–7,835	7,739
Poz-56285	759–761	C	7,045 ± 35	7,797–7,952	7,791–7,957	7,883

*El* *Erica* leaves; *Et* *Erica* twigs; *Bs* burned seed(s); *Bl* burned leaf fragment; *Lf* leaf fragment; *W* wood; *T* twigs; *P* periderm; *C* charred particles

^*^Rejected date

^a^ IntCal13,Reimer et al. (2013)

^b^ IntCal20,Reimer et al. (2020)

### Pollen, macrofossil and microscopic charcoal analysis

**Fig. 3:**
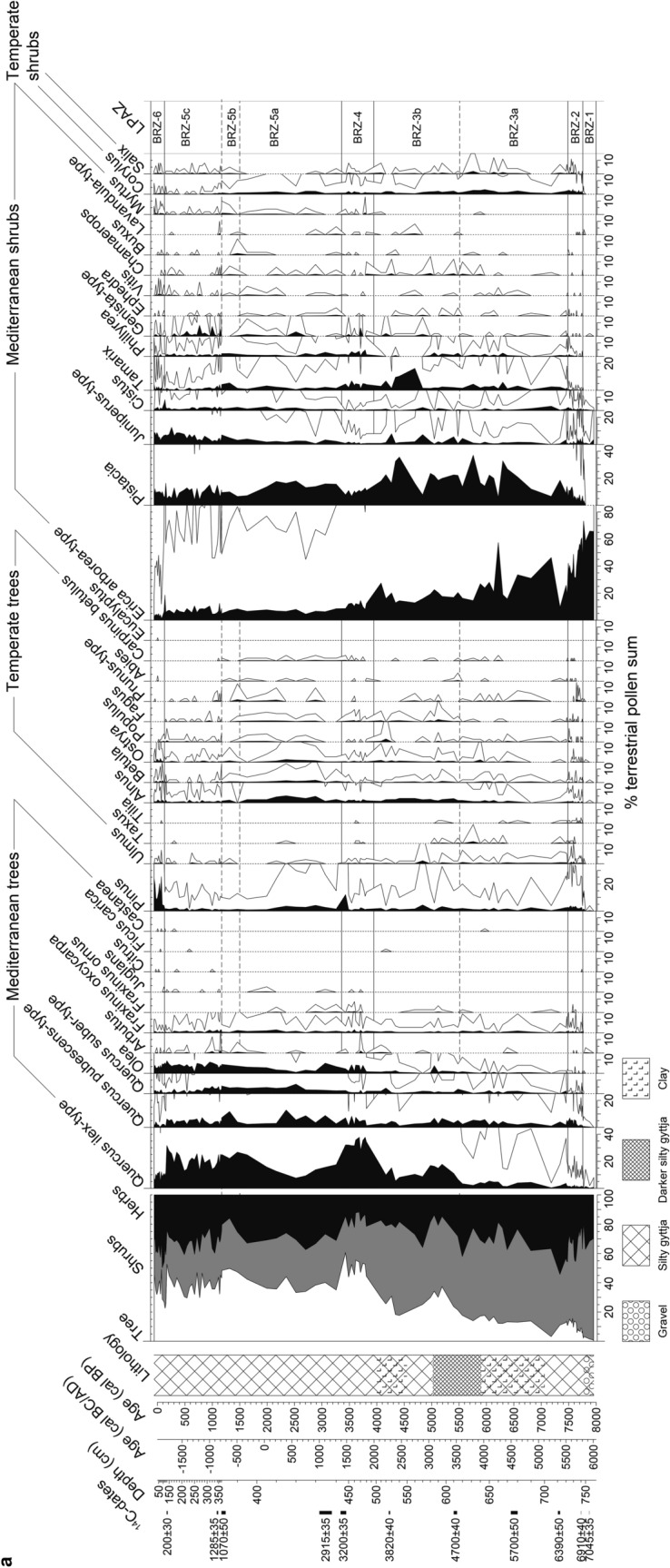

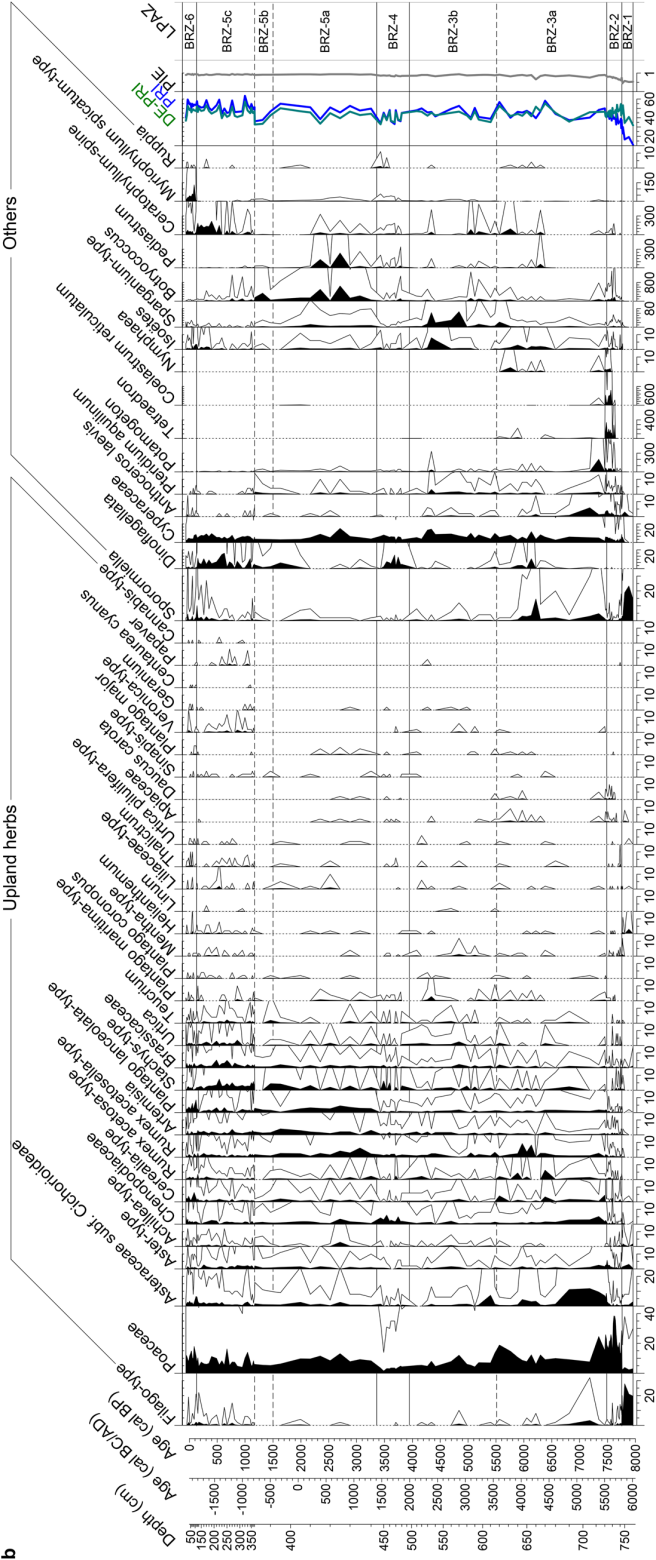
**a** Selected arboreal pollen percentages (AP), lithology and microscopic charcoal influx from Lago di Baratz (Italy). Empty curves show 10× exaggerations. LPAZ: local pollen assemblage zones. *Solid lines* show statistically significant boundaries. *Dashed lines* represent ecologically relevant boundaries (not statistically significant). **b** Selected non-arboreal pollen percentages (NAP) from Lago di Baratz along with wetland and water plants, Dinoflagellata, spores, palynological richness (PRI), detrended palynological richness (DE-PRI), and pollen evenness (PIE). *Empty curves* show 10× exaggerations. LPAZ: local pollen assemblage zones (see **a**)

**Fig. 4:**
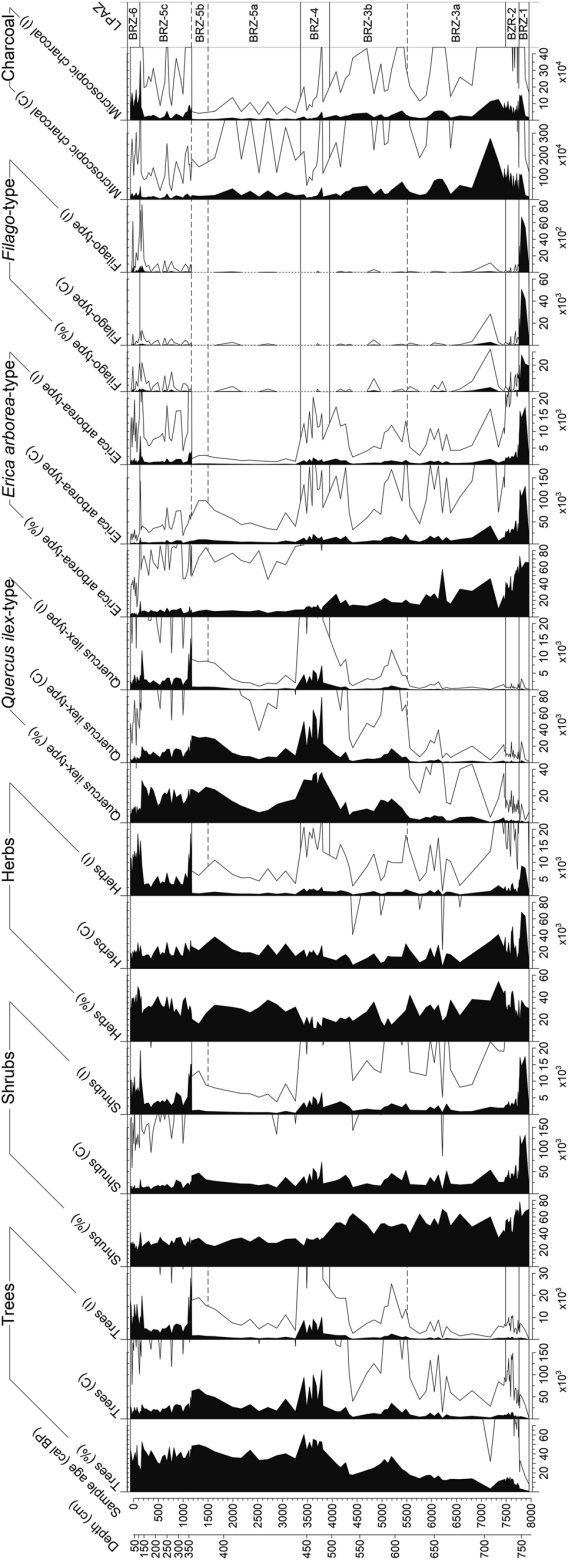
Pollen percentages, concentrations and influx of tree, shrub, herb sums and *Quercus ilex*-t., *Erica arborea*-t. and *Filago*-t. of core BRZ-D from Lago di Baratz (Italy), along with microscopic charcoal concentration and influx profiles. Curves show 10 × exaggerations. LPAZ: local pollen assemblage zones (see Fig. 3a)

A total of 122 samples of 1 cm^3^ were taken for pollen analysis. Six levels were found pollen-sterile and are therefore excluded. At an interval from 746 to 720 cm, 27 contiguous samples were obtained every centimeter for cross-correlation analysis during the period of the first *Erica* decline. Treatment followed standard methods for glycerine samples with HCl, KOH, HF and acetolysis (Moore et al. 1991). *Lycopodium* tablets (Stockmarr 1971) were added to calculate pollen concentration (grains cm^−3^) and influx (grains cm^−2^ yr^−1^). Identification of pollen types was based on keys and atlases (e.g. Moore et al. 1991; Reille 1992a; Beug 2004) as well as the reference collection at the Institute of Plant Sciences, University of Bern. The *Filago*-t. (t. = type) corresponds to the *Gnaphalium*-t. in Beug (2004; see Beffa et al. 2016). This pollen type is distinguishable from *Aster*-t. by its thick tectum as well as short echini (< 3–4 µm) and its rather small grain size (< 35 µm). The *Erica arborea*-t. includes *E. scoparia* (sensu Mateus 1989). The three *Quercus* pollen types *Q. pubescens*-t., *Q. ilex*-t. and *Q. suber*-t. were distinguished according to Beug (2004). A minimum of 500 terrestrial pollen grains per sample was counted. Statistically significant local pollen assemblage zones were delimited using optimal partitioning with minimal sum of squares (Birks and Gordon 1985) and the broken stick method (Bennett 1996), using the software R (R Development Core Team 2015). Subzone boundaries BRZ-3a/BRZ-3b, BRZ-5a/5b and BRZ 5b/5c were visually added because of their ecological relevance (Fig. 3). A minimum of 200 microscopic charcoal particles (≥ 10 µm) were identified at a magnification of 200× following Tinner and Hu (2003) and Finsinger and Tinner (2005). Charcoal concentrations (particles cm^−3^) and influx (particles cm^−2^ year^−1^) values were then calculated (Fig. 4) following the same approach as for pollen. Pollen diagrams (including microscopic charcoal results) were plotted using the program Tilia written by Eric Grimm.

**Fig. 5:**
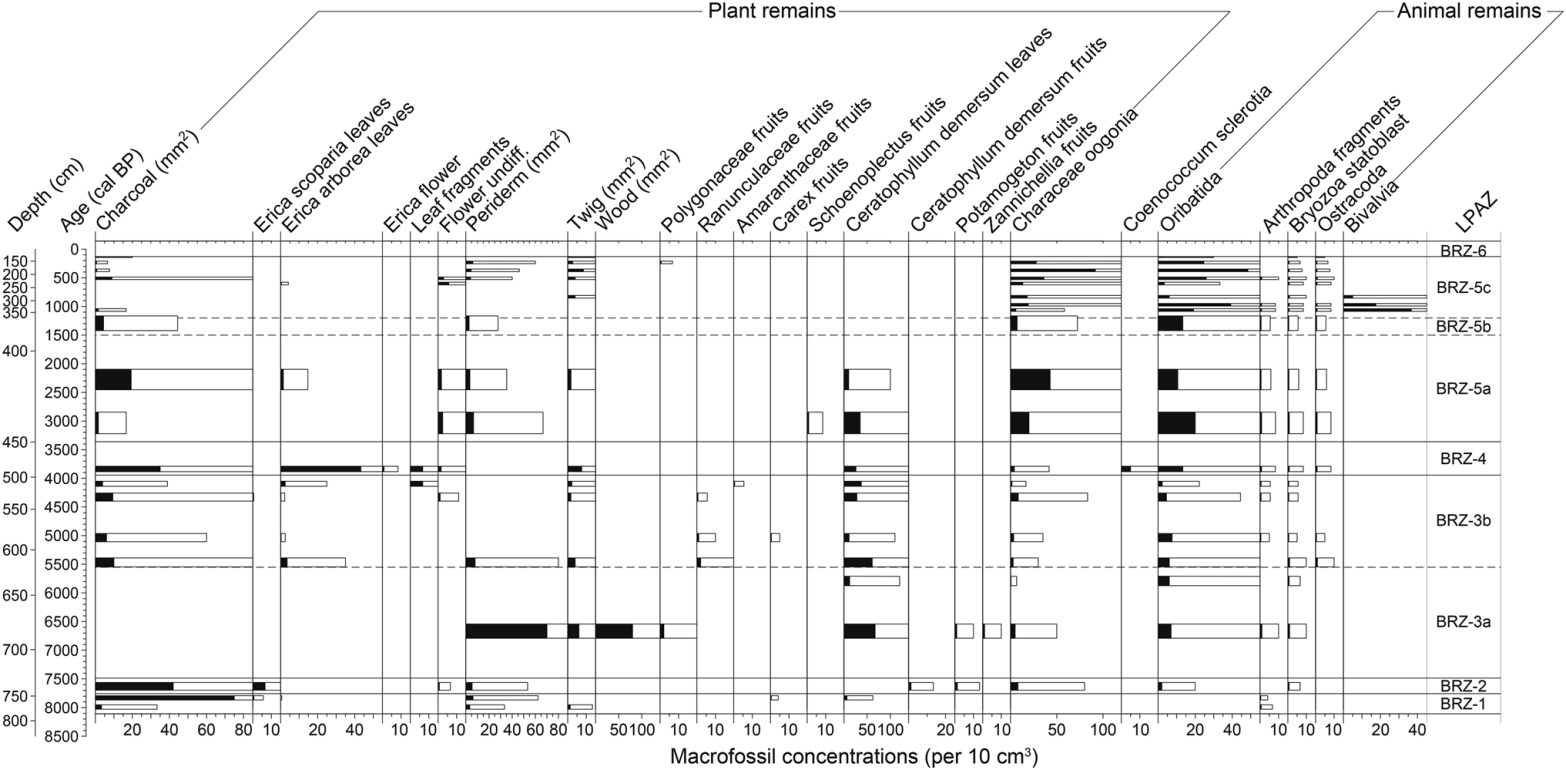
Plant-macrofossil concentration per 10 cm^3^ of Lago di Baratz. Empty bars show 5× exaggerations, *LPAZ* local pollen zones (analysts: Elias Zwimpfer, Giorgia Beffa)

Twenty-one two cm thick sediment samples, each with a volume of 10–24 cm^3^, were taken from between 118–780 cm for macrofossil analysis. Each sub-sample was then sieved with a mesh size of 200 μm. The extracted macrofossils were identified under a binocular microscope with plant morphological keys (e.g. Arrigoni 2006-2015; Cappers et al. 2006) and with the macrofossil reference collection of the Institute of Plant Sciences, University of Bern. Morphological discrimination between *E. arborea* and *E. scoparia* macrofossils followed Beffa et al. (2016). Macrofossil concentrations were standardized to 10 cm^3^ (Fig. 5).

### Geochemical analyses

**Fig. 6:**
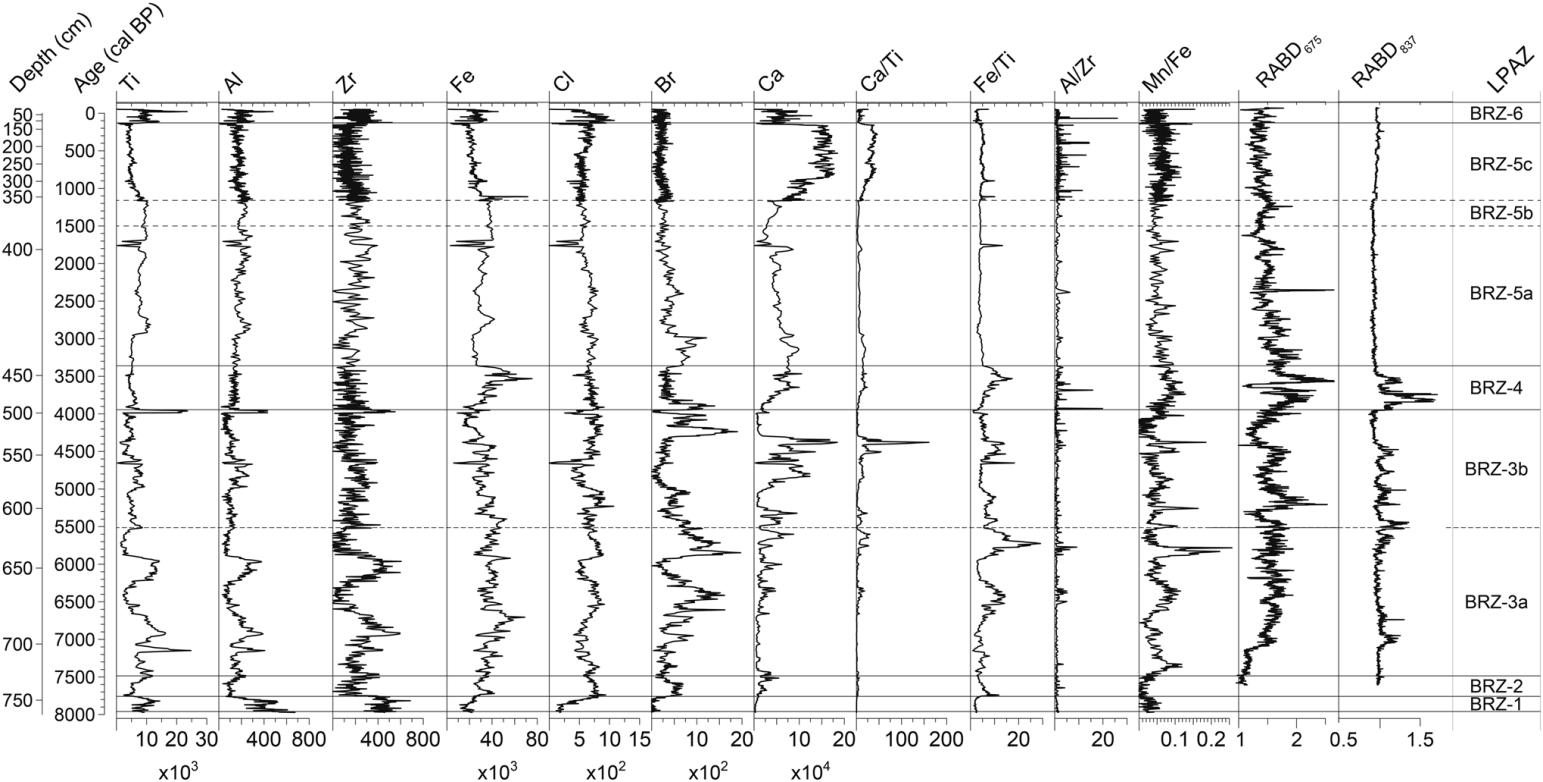
Elements and ratios from X-ray fluorescence (XRF) data counts and hyperspectral indices indicative of total aquatic productivity (RABD_675_) and hypolimnetic anoxia (RABD_837_). *LPAZ* local pollen assemblage zones (see Fig. 3a)

Elemental analysis was done at the Institute of Geological Sciences, University of Bern, with a Cr-tube equipped ITRAX XRF core scanner (Cox Ltd., Sweden), with a resolution of 5 mm and integration time of 10 s at 30 kV and 40 mA. All XRF data are quoted in counts. In our study, we focused on bromine (Br) as proxy for organic carbon and/or salinity (Ziegler et al. 2008; Bajard et al. 2016; Guevara et al. 2019), titanium (Ti), zirconium (Zr) and aluminum (Al) for sediment delivery from the watershed (Peterson et al. 2000; Haug et al. 2001), chlorine (Cl) for porosity in this brackish water body (Mongelli et al. 2013), and the ratios of manganese-iron (Mn/Fe) and iron–titanium (Fe/Ti) as indicators of redox-conditions at the sediment–water interface (Fig. 6). We assume that low Mn/Fe ratios are a result of lake-level highstands with a stratified water column and bottom-water anoxia that promote reductive dissolution of Mn-oxides in the water column and surface sediments and the formation of authigenic Fe-sulfides resulting in corresponding high Fe/Ti ratios (Naeher et al. 2013). High Ca and Ca/Ti values are interpreted as representing endogenic carbonate precipitation in the water column (Vogel et al. 2010).

Hyperspectral imaging (HSI) scans were performed at the Institute of Geography, University of Bern, using a Specim Ltd. Single Core Scanner equipped with a VIS-NIR PFE-CL-65-V10E hyperspectral camera (400–1,000 nm, Butz et al. 2015). Data normalization and post-processing followed Butz et al. (2015). We use the Relative Absorption Band Depth at 675 nm (index RABD_675_) as a proxy for total chlorophylls and their diagenetic products (Tchl) and, ultimately, aquatic primary production (Leavitt and Hodgson 2001) mainly by chlorophytes, diatoms and Chrysophyceae, among others. The Relative Absorption Band Depth at 837 nm (index RABD_837_) is diagnostic for bacteriopheophytin *a* and *b*, the degradation product of bacteriochlorophyll indicative of phototrophic purple sulfur bacteria PSBs (Yurkov and Beatty 1998; Butz et al. 2016). This suggests strong lake stratification and hypolimnetic anoxia with light at the top of the chemocline.

### Numerical analyses

To investigate biodiversity dynamics at Lago di Baratz, we calculated palynological richness (PRI, Birks and Line 1992) and the probability of interspecific encounter (PIE, Hurlbert 1971) as proxies for plant species richness and evenness, respectively. For PRI, rarefaction analysis was applied to a minimum pollen sum of 476 using the Vegan package (Dixon 2003; Oksanen et al. 2018) with the statistical software R (R Development Core Team 2015). We also evaluated evenness-detrended palynological richness (DE-PRI) to account for influences of palynological evenness on richness (Fig. 3b, Colombaroli and Tinner 2013).

**Fig. 7:**
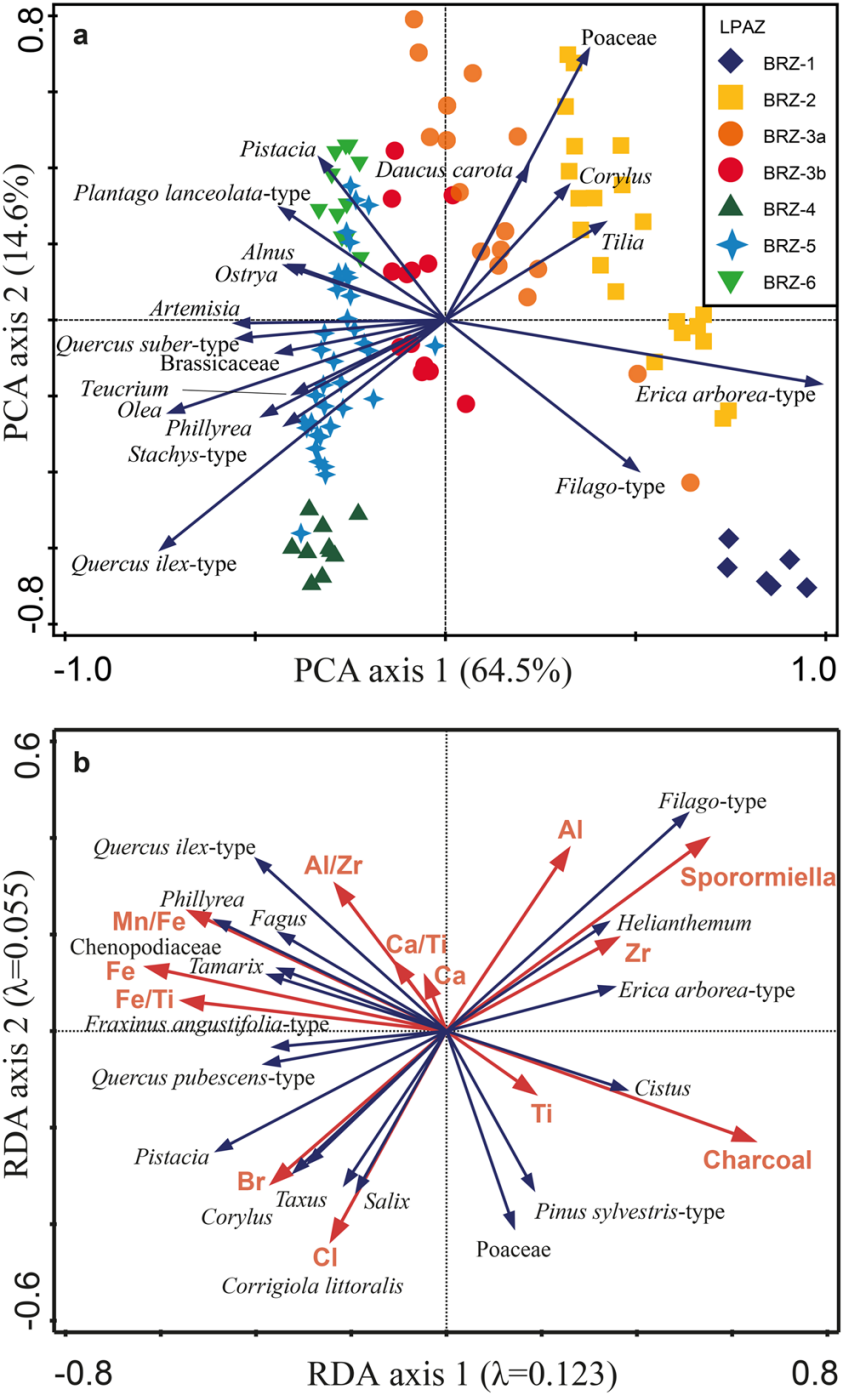
Ordination biplots. **a** PCA scatterplot of samples and selected taxa from Lago di Baratz, Italy. The first axis explains 64.5% of data variance, while the second axis explains 14.6%. Samples are grouped according to the local pollen assemblage zones (BRZ-1–6; see legend in Fig. 3). **b** RDA biplot, showing the relationship between selected plant species and explanatory variables. Two biotic variables include *Sporormiella* and microscopic charcoal influx. Eleven abiotic variables were obtained from elemental XRF analyses

In order to identify gradients in species and sample distribution, we applied ordination analyses on pollen percentages with the program Canoco 5.12 (Ter Braak and Šmilauer 2009). We selected linear response models of principal component analysis (PCA, Fig. 7a), and redundancy analysis (RDA, Fig. 7b), as suggested by the short gradient length of the first axis of detrended correspondence analysis (2.303 SD). For the RDA, we considered 13 environmental variables as explanatory factors. The two biotic variables include influx of microscopic charcoal, as a proxy for regional fires (Conedera et al. 2009) and influx of *Sporormiella* spores, a genus of fungi growing on animal dung, considered indicative of grazing herbivores (Davis and Shafer 2006; Etienne and Jouffroy-Bapicot 2014). In addition, we selected eleven abiotic variables from XRF elemental analyses (Ti, Al, Zr, Fe, Cl, Br, Ca, Ca/Ti, Fe/Ti, Al/Zr, Mn/Fe; see above). Canoco default RDA settings were used, including Monte Carlo permutation tests (Lepš and Šmilauer 2003) with *n* = 499 unrestricted iterations and sample age as a covariable.

**Fig. 8:**
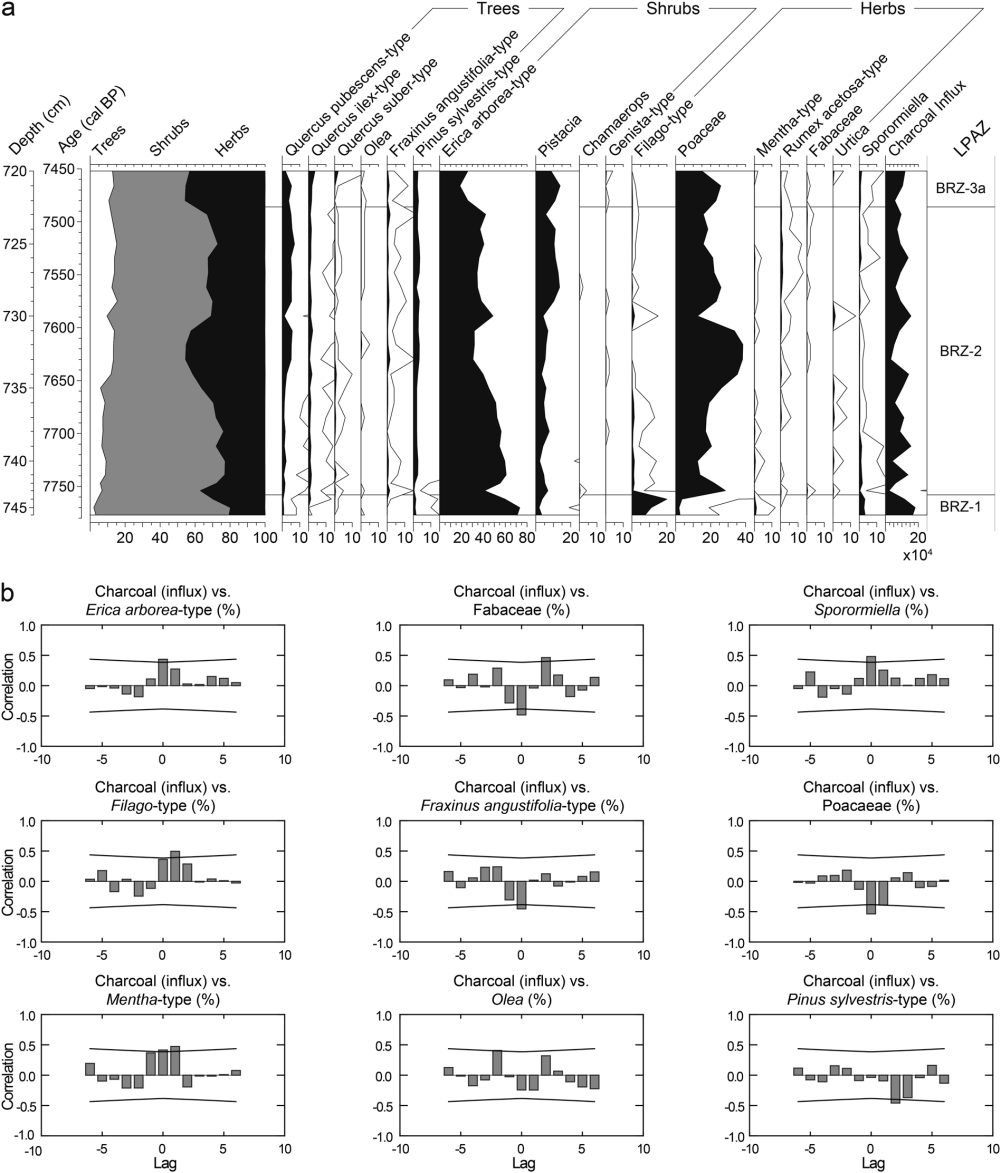
**a** Selected pollen percentages from the high-resolution section with contiguous sampling in 1 cm intervals (720–746 cm, 7,400–7,770 cal bp) from Lago di Baratz (Italy), along with microscopic charcoal influx. Empty curves show 10× exaggerations. **b** Cross-correlation diagrams of microscopic charcoal influx versus selected terrestrial pollen percentages for the high-resolution time-series, both variables were de-trended. 1 lag corresponds to 14 ± 1 years. The black lines mark the significance level of the correlation coefficient (*P* = 0.05)

To infer the importance of fire for vegetation dynamics at Lago di Baratz, we calculated cross-correlations (Green 1981) on the 27 contiguous samples (720–746 cm; 7,780–7,450 cal bp) with an interval-sample age of 14.2 ± 1.0 cal years. Charcoal influx and pollen percentages were first linearly detrended to achieve stationarity and successively compared and plotted using the software Mystat 12.02.00 (Systat Software Inc 2007) with 6 lags (in both time directions), each lag corresponding to 14 years (Fig. 8).

## Results and interpretation

### Chronology, sedimentology, element geochemistry and pigment analyses

The chronology is built on 12 AMS dates on terrestrial plant macrofossils (Table 3). The depth-age model shows that the period 8,000 to 2,500 cal bp is chronologically well constrained, while the reliability declines during the period 2,500 to 200 cal bp due to inversions of dates (Fig. 2). Two inflection points in the age-depth model at ca. 3,500 and 1,200 cal bp correspond to sedimentary changes as revealed by XRF analyses (marked decline of Fe, increase of Ca respectively, see below). We therefore conclude that the changes in sedimentation rates are caused by depositional dynamics and not by the (arbitrary) position of the radiocarbon dates. The sediments consist of coarse gravel at the bottom of the succession (802–743 cm, 8,200–7,750 cal bp, Table 2). In this section Ti, Al and Zr are high, suggesting a predominant deposition of coarse detrital siliciclastics, possibly with low porosity (Cl values are lowest, Fig. 6). Interestingly other coastal sedimentary records in Sardinia also start at a comparable age (Di Rita and Melis 2013; Beffa et al. 2016; Melis et al. 2017). We assume that Lago di Baratz originated from a Holocene sea-level transgression at around 8,200 cal bp resulting in rising groundwater levels and landward sand-dune movement, damming the water from freshwater streams (Colombaroli et al. 2007; Beffa et al. 2016).

At 746 cm (ca. 7,750 cal bp), the sediment shifts upcore from sandy gravel to silty gyttja (Table 2, Fig. 3) and indicators for erosion (Ti, Al and Zr, Fig. 6) decrease sharply, suggesting a sustained lake-level highstand with finer sediments deposited at the coring site. As a consequence, sedimentation rates stabilize between 760 to 450 cm (7,850–3,500 cal bp) at ca. 0.6 mm per year, resulting in 14 years per 1 cm (i.e. thickness of pollen samples). A sharp increase in Br around 670 cm (6,500 cal bp) and relatively high values in Cl (Fig. 6) suggest more organic production in the lake when porosity increased, and/or an increase in water salinity. Changes in the Mn/Fe ratio between 722 cm (7,500 cal bp) and 495 cm (3,950 cal bp) indicate that redox conditions were highly variable, possibly due to fluctuating lake levels and the resulting change in water-column stratification. Peaks of Ti, Al and Zr around 7,200 and 6,100 cal bp point to a stronger influx of detrital siliciclastics to the centre of the lake. Between 641 and 583 cm (5,900–5,000 cal bp), dark silty gyttja combined with rather stable values in detrital indicators (Ti, Al, Zr) and a relatively high Fe/Ti ratio suggests calm, profundal conditions in the lake. A marked decrease in sedimentation rates (Figs. 2, 3) suggests rather stable catchment conditions between ca. 450 and 382 cm (ca. 3,500–1,200 cal bp). Above 382 cm (ca. 1,200 cal bp), sedimentation rates increase to reach only ca. 2–3 years per cm (i.e. thickness of pollen samples). This increase in sedimentation rates was mainly caused by increased authigenic carbonate deposition (elevated Ca and Ca/Ti ratios). During the last 130 years of the record, Ti, Al and Zr show high values, suggesting increased input of detrital siliciclastics, possibly as a result of increased land use, river management and/or lowered lake level. Reduced Ca values may point to less evaporation (or more rainfall) during this period. Taken together, the combined geochemical evidence suggests that during the past 8,000 years, detrital input, lake productivity and lake level varied considerably at multidecadal to centennial scales; yet there is no evidence for a persistent Mid to Late Holocene multi-millennial trend to lower lake levels (e.g. Zr, Ca, Mn/Fe ratio), suggesting that aridity did not increase during the Late Holocene.

The aquatic primary production (index RABD_675_) increased substantially around 7,000 cal bp and remained high with marked decadal- to centennial-scale fluctuations until ca. 4,300 cal bp (Fig. 6). Subsequently primary productivity peaked between 4,000–3,500 cal bp (BRZ-4). A gradual decrease in aquatic primary production followed until 2,500 cal bp, when it stabilized at relatively low levels (compared with the Mid Holocene) until today. However, decadal-centennial-scale variability persisted. Periods of multidecadal-long hypolimnetic anoxia (index RABD_837_) with substantial amounts of sedimentary bacteriopheophytins Bphe (indicative of purple sulfur bacteria) occurred around 7,000 cal bp and four times between 5,500 and 4,500 cal bp. A period with sustained anoxic and possibly meromictic conditions occurred between 4,000–3,500 cal bp, when pollen suggests rather closed forest conditions (see BRZ-4 below, *Quercus ilex* maximum, arboreal pollen ca. 80%). Similar to the pollen profile, this 500 years-long period does show a structure in the Bphe with two peaks. After 3,500 cal bp, no prolonged anoxic periods are observed anymore, when the pollen record suggests major forest clearances in the catchment.

### Pollen and macrofossil inferred vegetation history

We identified six statistically significant local pollen assemblage zones (LPAZ, Fig. 3). Subzone boundaries (BRZ-3a/b; BRZ-5a/b, BRZ-5b/c) were added visually to refine the subdivision where the duration of the zone was long (ca. 3,000 years). The pollen sequence starts at 770 cm (ca. 8,000 cal bp). Overall, pollen percentages, concentrations and influx are in very good agreement, showing that the percentage calculations are not affected by internal distortions (Faegri and Iversen 1989; Fig. 4). During the past 1,200 years however, pollen influx (trees, shrubs, herbs) departs from pollen percentages and concentrations. This is probably a consequence of the massive increase in sedimentation rates and the resulting in-washed pollen or, alternatively, of chronological uncertainties. Similarly, charcoal concentrations and influx are in good agreement until charcoal influx increases drastically ca. 1,200 cal bp (Fig. 4). One major issue when reconstructing vegetation history at sites where *Erica* species play a major role is that they can grow as both medium-sized trees (up to ca. 20 m), or shrubs. Thus, they have the potential to form both, forests and shrublands (see discussion in Beffa et al. 2016). In this study we assigned the pollen of *Erica* to shrubs, because this is the prevalent growth form of *Erica* species in the Mediterranean today. However, this life trait might have been affected by anthropogenic disturbance. Hence, we cannot exclude that *Erica arborea* and *E. scoparia* were growing as trees forming rather closed forests, as still observed in some areas of the Mediterranean and elsewhere in their distribution ranges (see Beffa et al. 2016 and references therein).

BRZ-1: 8,000–7,750 cal bp. High *E. arborea*-t. percentages (always over 60%, with peaks of 73%, Fig. 3a) and macrofossil finds (Fig. 5) of *E*. *scoparia* suggest that *Erica* woodlands dominated the vegetation around the lake. The high abundance of *Filago*-t. pollen (> 20%) can be explained by the ecology of the taxon, which is abundant on dry meadows as well as on pure sand or sandy soils (Beffa et al. 2016; Pignatti et al. 2017-2019, Fig. 3b). Besides *Filago*-t., other herbaceous pollen types (e.g. Poaceae*, Aster-*t. and Brassicaceae) show the presence of open communities. *Sporormiella* fungal spores reach highest values (up to 23%), suggesting the presence of mammals exploiting the freshwater lake. Scattered occurrences of Cerealia-t. pollen in this zone suggest the occurrence of cultivated cereals around the onset of Neolithic agriculture. An attribution of Cerealia-t. pollen to cultivated instead of related wild grasses is plausible, given that einkorn and emmer caryopses were found in Sardinian archaeological sites during the period 8,000–7,500 cal bp (Ucchesu et al. 2017b). Low abundances of evergreen and deciduous arboreal taxa (e.g. *Quercus ilex*-t., *Q. pubescens*-t., *Arbutus*, *Olea*, *Pinus*, *Alnus*, *Ulmus*, *Ostrya*, *Cistus*, Cupressaceae—including *Juniperus—*and *Corylus*) show that these taxa were rare in the study area. Spores of the moisture-demanding moss *Anthoceros laevis* as well as pollen of thelmatic Cyperaceae (< 7%), suggest the presence of swampy areas at the lake shores. Diversity analyses indicate that species richness was rather low, although DE-PRI values show that very low PRI may mainly result from low palynological evenness (caused by the dominance of *E. arborea*-t. and *Filago-*t.). Both microscopic charcoal particle influx (> 150,000 particles cm^−2^ yr^−1^) and macroscopic charcoal concentrations (80 mm^2^/10 cm^3^) reach high values towards the end of the zone, suggesting high regional fire activity and local fire occurrence at around 7,800 cal bp, when *E. arborea*-t. and *Q. ilex*-t. reached their highest (74%) and lowest (< 1.4%) Holocene abundances, respectively.

BRZ-2: 7,750–7,500 cal bp. This LPAZ is characterized by the decline of *E. arborea*-t. from ca. 60 to 40% (Figs. 3a and 8). Macrofossil finds show that the main *Erica* species was still *E. scoparia* (Fig. 5). The vegetation was rather open as evidenced by arboreal pollen (AP) around 60–70%. The pollen data suggest that oaks (*Q. ilex*-, *Q*. *pubescens*- and *Q*. *suber*-t.), started to expand together with some shrubs (*Pistacia*, Cupressaceae and *Cistus*). We assume that most *Pistacia* pollen derived from evergreen *P. lentiscus* (mastic), a characteristic species of the thermo-mediterranean maquis that is still abundant today around the site. Pollen from other woody taxa such as *Fraxinus ornus*, *F. angustifolia*-t., *Ephedra*, *Tamarix*, *Phillyrea*, *Chamaerops humilis* and *Buxus* occurs for the first time, suggesting their scarce presence in local plant communities. The composition of local herb- and grass-dominated assemblages shifted, specifically *Filago*-t. declined and Poaceae increased, probably in response to soil formation resulting in less sandy, more developed soils. Increases of Cerealia-t., *Rumex*, *Plantago lanceolata-*t. and *Urtica*, along with a moderate increase of Chenopodiaceae, *Artemisia* and Brassicaceae point to agricultural activities around the lake. *Sporormiella* spores drop to less than 2% pointing to decreasing grazing, perhaps of wild animals, close to the lake. Several green algae including *Tetraedron*, *Coelastrum* and *Botryococcus* occur, possibly indicating nutrient enrichment of the lake (Jankovská and Komárek 2000)*. Isoetes* spores, *Sparganium*-t. and *Potamogeton*-t. pollen, and *Ceratophyllum* spines also occur in this zone, documenting freshwater conditions (Stevenson et al. 1993; Beffa et al. 2016). PRI and DE-PRI increased during this zone (Fig. 3b), suggesting that vegetation became more diverse. Microscopic charcoal influx values fluctuate between 50,000 and 100,000 particles cm^−2^ yr^−1^. In comparison to the previous LPAZ, they suggest slightly reduced fire activity in the region, while high macroscopic charcoal concentration (40 mm^2^/10 cm^3^) points to the occurrence of fires close to the lake.

BRZ-3: 7,500–3,950 cal bp. The most remarkable feature of this LPAZ is the steady increase of AP (from ca. 50 to 80%) and the increase of *Q. ilex*-t. (from 3 to ca. 20%), which suggest a marked expansion of evergreen oak woodlands or forests. The pollen influx values of *Q. ilex*-t. increase from ca. 100 to 2,000 pollen grains cm^−2^ yr^−1^, showing that the stands became rather dense (Tinner et al. 2009; Fig. 4). First finds of *E. arborea* macrofossils testify that the species became gradually more important than *E. scoparia* during this zone, which is lacking in the macrofossil record after 7,500 cal bp (Fig. 5). PRI and DE-PRI were relatively high (40–50 types/sample) with peaks during periods with increased non-arboreal pollen (NAP). In general, microscopic charcoal-inferred regional fire activity declined after 7,000 cal bp (from 150,000 to 50,000 particles cm^−2^ yr^−1^), when evergreen oak forests started to expand.

The decrease of *E. arborea*-t. from 40 to < 10% suggests that at the beginning of the subzone BRZ-3a, between 7,500 and 7,300 cal bp, *Erica* woods strongly declined, while *Pistacia* shrubs or small trees spread together with herbs such as Poaceae and Cichorioideae. Around 7,200 cal bp, regional fire activity increased markedly (microscopic charcoal 130,000 particles cm^−2^ yr^−1^) and *Erica* woodlands re-expanded together with *Cistus*, *Tamarix*, *Filago*-t*.*, Chenopodiaceae and Cichorioideae, while other woody species such as *Q. ilex*-t., *Fraxinus angustifolia*-t. and *Pistacia* were reduced. After 7,000 cal bp, *E. arborea*-t. decreases, while *Q. ilex*-t. moderately re-increases along with other species of the thermo-mediterranean forest (e.g. *Q. pubescens*-t. and *Olea*) and maquis vegetation (*Pistacia* up to 37%, *Phillyrea*, Cupressaceae). Cerealia-t. (4.5%) and secondary anthropogenic disturbance indicators (*Rumex*, *Plantago lanceolata*-t*.*, Brassicaceae, *Urtica*, *Artemisia*) increase, pointing to intensifying agriculture around the lake. *Sparganium*-t., *Nymphaea* and *Isoetes* pollen or spores increase as well, suggesting changing lake-level conditions and/or expansion of wetlands. Short-term recoveries of *E. arborea*-t. at the expense of *Pistacia* and *Q. ilex*-t. are superimposed on the long-term expansion trend of evergreen oak forests with mastic understorey and/or mastic maquis such as around 6,400 cal bp, when regional fire incidence was high.

The pollen data suggest that in subzone BRZ-3b (after 5,500 cal bp) *Q. ilex* continued spreading (up to 35%) and *Olea* became more abundant, when according to the microscopic charcoal record, regional fire incidence further declined (Fig. 3a). The water plants *Sparganium*-t., *Ceratophyllum* and *Botryococcus* expanded in the lake, most likely in response to lake level increases, as suggested by our palaeoenvironmental proxies (dark silty gyttja, stable values in detrital indicators Ti, Al, Zr and relatively high Fe/Ti ratio). Around 4,700 cal bp, pollen abundance of the salt-tolerant marsh shrub *Tamarix* (Pignatti 2005; Pignatti et al. 2017-2019) peaked (reaching 17%), possibly in response to a transient lake-level drop (Ca peak, Fig. 6) and/or a salinity increase. In agreement, when *Tamarix* stands declined around 4,500 cal bp, salt-intolerant water plants such as *Isoetes* and *Potamogeton* (Pignatti 2005) spread. Subsequently *Q. ilex* forests and *Pistacia* shrublands expanded massively, when regional fire activity was rather moderate (< 40,000 particles cm^−2^ yr ^−1^).

BRZ-4: 3,950–3,350 cal bp. The dominance of AP (> 80%) suggests the presence of closed forest vegetation dominated by *Q. ilex*-t. together with *Olea* and *Q. pubescens*-t. Co-dominant shrubs were *Pistacia, E. arborea*-t*., Phillyrea* and *Tamarix,* while other characteristic taxa of the thermo-mediterranean vegetation such as *Q. suber*-t., *Chamaerops humilis, Cistus*, *Myrtus communis*, *Phillyrea*, *Buxus* (quite likely *B. balearica*), *Lavandula*-t. and *Genista* were less abundant. First findings of *Juglans* pollen point to the introduction of this tree on the island. Pollen of salt-tolerant *Ruppia* and Chenopodiaceae (Pignatti 2005; Pignatti et al. 2017-2019) increase together with a prominent peak of dinoflagellates, pointing to lake stratification with saline bottom and fresh surface water, given that salt-intolerant *Isoetes* was also present (Beffa et al. 2016). PRI and DE-PRI inferred biodiversity diminished only slightly and microscopic charcoal-inferred regional fire activity continued declining during this period (ca. 10,000 particles cm^−2^ yr^−1^).

BRZ-5: 3,350–150 cal bp: This zone is characterized by the disruption of forests and the expansion of open vegetation, as indicated by AP dropping to 60–70%. *Quercus ilex* and *Pistacia* dominated local woodlands. PRI and DE-PRI reach their highest Holocene values (> 60 taxa per sample), suggesting a species-rich mosaic of forests, shrublands, grasslands with pastures and extensive croplands. In the youngest part of this zone, microscopic charcoal influx suggests an increase of regional fire activity, which is however not mirrored in the concentration values (Fig. 4).

In BRZ-5a (3,350–1,500 cal bp), *Q. ilex*-t. declines to < 10% at ca. 2,600 cal bp. *Olea* pollen percentages rise between 3,350 and 3,000 cal bp together with *Q. suber*-t. and *Q. pubescens*-t. At the same time, many herbs (in particular, Poaceae) increase, indicating expansions of open land. Increases in pollen abundances of weeds and crops such as *Plantago lanceolata*-t*., Rumex acetosella*-t. and Cerealia-t. show that this opening was likely related to forest clearances for arable and pastoral farming. Also, the peaks in pollen percentages of fruit trees such as *Juglans* and *Prunus*-t. are striking. *Botryococcus* and *Pediastrum* green algae significantly increase while dinoflagellates almost disappear from the record, suggesting a shift in water quality. Around 1,700–1,500 cal bp, evergreen oak forest recovered mainly at the expense of *Pistacia* shrublands, with *Q. ilex*-t. and *Olea* reaching percentages of 27 and 8%, respectively. The open land share remained rather stable (NAP = 30–40%). In the subzone BRZ-5b (1,500–1,200 cal bp), *Q. ilex*-t. remains important (ca. 20%) and AP reaches ca. 80%, suggesting a last major expansion of woodlands and shrublands, probably in response to land abandonment (Cerealia-t., *Rumex acetosa*-t., *Urtica* minima) during the early medieval period. During subzone BRZ-5c (1,200–150 cal bp), open land gradually re-expanded and fire activity increased (Fig. 3a). However, coeval increases of tree, herb and shrub influx, with a shape comparable to charcoal influx, suggest that influx values are generally affected by high sediment accumulation. High accumulation rates were likely caused by endogenic carbonate precipitation in the water column (high Ca. Vogel et al. 2010; Fig. 6), thus it is unlikely that increased microfossil delivery was caused by erosion of terrestrial soils (see also declining Ti values). Instead, carbonate precipitation may indicate higher evaporation and/or lowered lake levels during the period 1,200–150 cal bp, which may have resulted in higher pollen supply from exposed (calcareous) shore sediments. Several herb taxa peaked (e.g. *Filago*-t., Brassicaceae, *Urtica, Veronica*-t.) and *Sporormiella* increased, suggesting an expansion of pastoral farming that was beneficial to diversity (high PRI and DE-PRI). While salt-tolerant *Tamarix* shrubs declined, Dinoflagellata cysts and *Ceratophyllum* spines show highest values in this subzone.

BRZ-6 (150 cal bp until present): *Q. ilex*-t*.* and AP decrease markedly, while *Pinus* and NAP increase, mainly as a result of pine plantations to stabilize the dunes and further creation of open land (e.g. Cichorioideae, Poaceae, Cerealia-t.) for pastoral and arable farming. Introduced *Eucalyptus* shows first pollen occurrences. The increase of pollen of the water plant *Myriophyllum spicatum* may result from recent efforts to raise the water table (Giadrossich et al. 2015). PRI and DE-PRI remained stable or slightly declined towards the present, however, the resolution of this most modern zone is insufficient to address potential negative effects of intense land use on biodiversity. Microscopic charcoal particles increase to highest values in the record, up to 350,000 particles per cm^−2^ yr^−1^, which is to some extent mirrored in the concentration values. We assume increased regional fire incidence, perhaps comparable to that of 8,000–7,000 years ago.

### Time-series analyses

We applied cross-correlations to understand the response of plant taxa to fire in the time interval 7,770–7,450 cal bp, when *Erica* species (mostly *E. scoparia,* see macrofossils in Fig. 5) collapsed for the first time (from 70 to 20%, Figs. 3 and 8). The striking and rather rapid decline is also recorded in pollen concentration and influx values, from ca. 18,000 to < 1,000 pollen grains cm^−2^ yr^−1^ (Fig. 4). *E. arborea*-, *Filago*- and *Mentha*-t. show significant positive correlations with charcoal influx, as does *Sporormiella*. In contrast, *Fraxinus angustifolia*-t., Fabaceae and Poaceae display significant negative correlations (Fig. 8). The few *Q. ilex*-t. pollen grains (< 5%) show no significant correlation with fire (data not shown). These results suggest that *E. arborea*-t. was promoted by fire disturbance. Fire also fostered open xerophytic or open land vegetation, specifically the striking *Filago*-t. abundance. The positive correlation of fire with *Sporormiella* is best explained by copious animal presence during the periods of increased fire activity. This finding suggests that through the disruption of woodlands and the promotion of grasslands (herb pollen increase, Fig. 8), fire disturbance had beneficial effects on mammal populations. In the absence of a clear link between charcoal and agricultural indicators, we assume that both fire incidence and animal presence were natural or quasi-natural, likely in response to dry conditions causing high fire activity and gathering of animals close to the freshwater sources (Beffa et al. 2016).

### Ordination analyses

PCA axes 1 and 2 explain 64.5% and 14.6% of the variance in the dataset, respectively (Fig. 7a). PCA axis 1 spans a gradient from *E. arborea*-t. and *Filago*-t. to *Q. ilex*-t., *Q. suber*-t. and *Olea*, thus likely reflecting light availability and/or disturbance in pristine forested environments, with *E. arborea*-t. and *Filago*-t. being light-demanding, drought-resistant and fire-tolerant taxa, and *Q. ilex*-t., *Q. suber*-t. and *Olea* being more shade-tolerant and less drought- and fire-resistant (Beffa et al. 2016). PCA axis 2 may reflect agricultural activities, as it is mostly controlled by a gradient from anthropogenic indicators and grassland taxa such as *Plantago lanceolata*-t., *Daucus carota* and Poaceae to late successional shade-tolerant and long-lived taxa such as *Q. ilex*-t. Herbaceous taxa that do not fit this pattern, such as the pioneer *Filago*-t., were dominant when human impact was negligible (BRZ-1).

According to RDA analysis all environmental variables together explain 41.3% of the variance in the pollen data. Among the most important variables are the ratio Mn/Fe (10.9%), *Sporormiella* influx (9.6%) and microscopic charcoal influx (4.5%). More specifically, *Sporormiella*, charcoal, Zr, Al and Ti have high axis 1 scores, suggesting higher rates of detrital inflow during periods of high grazing pressure or fire activity (Fig. 7b). Correlated with those disturbance indicators are *E. arborea*-t., *Filago*-t. and *Cistus*, species that are well adapted to fire and grazing. This pattern points to human impact driving both environmental (XRF, charcoal) and biotic change (pollen, spores). On the other side of the gradient, woody taxa from Mediterranean forest and maquis such as *Q. ilex*-t., *Q. pubescens*-t., *Pistacia* and *Phillyrea* are grouped together with Fe, Br and Mn/Fe, indicating stable and productive conditions, likely under low human impact.

## Discussion

### Environmental and climatic conditions and their effect on vegetation and fire regime

Mediterranean sea-level reconstructions show that sea-level rise culminated around 9,000–8,000 cal bp (Brisset et al. 2018; Vacchi et al. 2018). The Mediterranean Sea rose 15 m between 9,000 and 8,000 cal bp, while in the following 8,000 years the further increase was only ca. 5 m to reach modern conditions (Brisset et al. 2018; Surdez et al. 2018; Vacchi et al. 2018). We assume that the Early Holocene transgression caused the formation of lagoons in Sardinia, with the sea-level rise promoting the rise of groundwater tables as well as the retention of freshwater in the retro-dunal depressions (Surdez et al. 2018). Today water salinity is moderate at Lago di Baratz, with Na reaching concentrations of 350–400 mg/L and Cl 620–660 mg/L, allowing freshwater animals and plants to thrive (Mongelli et al. 2013). XRF (e.g. Cl) and pollen data (e.g. *Potamogeton*, *Myriophyllum spicatum*-t.) suggest that, with the exception of minor salinity oscillations, freshwater conditions prevailed in Lago di Baratz during the past 8,000 years (Figs. 3b and 6), which is comparable to the situation at the eastern Sardinian lagoon Sa Curcurica (Beffa et al. 2016). In the catchment of Lago di Baratz, lake levels and/or erosional activity oscillated markedly from 8,100 to ca. 5,500 cal bp, as documented by the XRF data (Ti, Al, Zr; Fig. 6). Fluctuations of these elements may have originated from recurrent flood events, perhaps related to former lake inlets that are inactive today but still visible in the landscape. Given the competitiveness of *Erica* on wet sandy soils, we assume that together with recurrent fires, floods created environments suitable for the two species, for example repeated deposition of vast sand banks*.* The flood events may have been the result of heavy rainfall at higher elevations north-east of the lake, perhaps as a consequence of marked seasonal climates prior to 7,000–6,000 cal bp (Frisia et al. 2006; Curry et al. 2016; Budsky et al. 2019). However, these disturbance-enhanced Mid Holocene environmental and vegetation patterns subsequently ended from 5,500 to 4,000 cal bp, when evergreen oak forests stabilized the slopes and soils around the site, and fires became less frequent.

Vegetation conditions in Sardinia prior to 8,200 years ago can be inferred from the inland pollen and anthracological record of Grotta Corbeddu (185 m a.s.l., North-East Sardinia, Fig. 1). This multiproxy record suggests that during the Early Holocene, thermo-mediterranean vegetation on soils deriving from calcareous bedrock was dominated by pine-olive-evergreen oak woodlands (Kalis and Schoch 2019). Deciduous oaks were probably growing at higher altitudes, above ca. 1,000 m a.s.l. The expansion of Ericaceae (most likely *Erica*) occurred at around 9,000–8,000 cal bp, when the entrance to the cave collapsed (Kalis and Schoch 2019). Evidence from neighbouring Corsica shows that pine forests dominated the vegetation at intermediate altitudes (Lac de Creno, 1,310 m a.s.l.) during the Early Holocene (Reille et al. 1997), while the subsequent mass expansion of *E. arborea* occurred at the onset of the Mid Holocene period, i.e. around 8,200 years ago (Reille 1992b), which is in good agreement with the Sardinian record of Grotta Corbeddu. It is difficult to assess the causes and processes that led to this high dominance of *Erica* species as seen in Sardinia (e.g. Beffa et al. 2016; Melis et al. 2018) and Corsica (Reille et al. 1997; Revelles et al. 2019) at the onset of the Mid Holocene (ca. 8,000–7,000 cal bp). This feature might be a legacy of the environmental and ecological conditions during the ice age, when both islands were connected by a land bridge. Indeed, a comparable Mid Holocene dominance of *Erica* species in Corsica and Sardinia did not occur in mainland Italy and Sicily (e.g. Kelly and Huntley 1991; Magri 1999; Magri and Sadori 1999; Allen et al. 2002; Drescher-Schneider et al. 2007; Noti et al. 2009; Tinner et al. 2009; Calò et al. 2012; Sadori et al. 2013). Sardinia’s geographical isolation may have represented an obstacle to colonization of the island by plant species at the end of the last glacial maximum, which would have provided competitive advantages to those species that survived locally. In this regard, temperate European trees, such as *Abies alba*, *Acer pseudoplatanus*, *A. platanoides*, *Quercus cerris*, *Q. petraea*, *Q. robur*, *Fagus sylvatica*, *Carpinus betulus*, *Tilia cordata*, *T. platyphyllos* as well as boreal *Betula pubescens* and *B. pendula* currently lack natural stands in Sardinia (Arrigoni 2006-2015; Pignatti et al. 2017-2019). However, pollen of *Abies*, *Fagus*, *Carpinus*, *Tilia* and *Betula* occurs regularly in the Lago di Baratz record (Fig. 3). Given that the study site is in the thermo-mediterranean vegetation belt, we are unable to resolve whether these taxa were present in the Sardinian highlands during the Holocene (e.g. in the mountains within the wider pollen catchment of ca. 30–50 km distance, Conedera et al. 2006) or if the pollen (Fig. 3) came from stands in neighbouring Corsica. However, support for the hypothesis that the isolated location of islands is ecologically important, derives from the observation that several Mediterranean islands feature unique post-glacial vegetation histories that are lacking on other islands or on the Mediterranean mainland (see Gambin et al. 2016; Burjachs et al. 2017). This palaeoecological finding may explain the presence of island-specific shifts of niches (Goedecke et al. 2020).

**Fig. 9:**
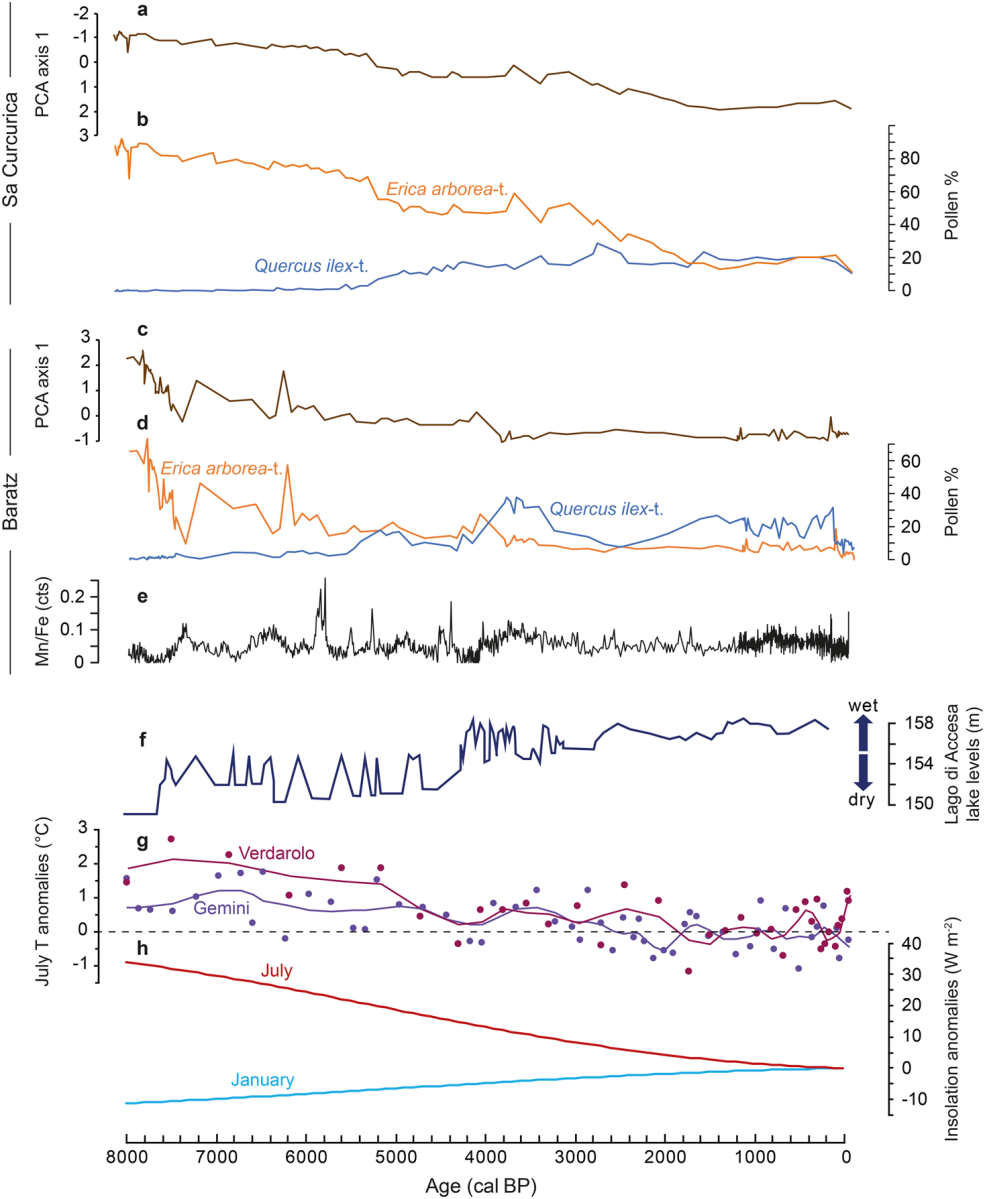
Comparison of ordination analyses, biotic and abiotic proxies from Lago di Baratz and Stagno di Sa Curcurica combined with different local and regional climate records: **a** principal component analysis (PCA) axis 1 sample scores from Stagno di Sa Curcurica (Beffa et al. 2016), as well as **b** pollen influx of *Erica arborea*-t. (*orange*) and *Quercus ilex*-t. (*dark blue*); **c** principal component analysis (PCA) axis 1 sample scores from Lago di Baratz, as well as (d) pollen influx and (e) pollen percentages of *E. arborea*-t. (*orange*) and *Q. ilex*-t. (*blue*); (f) Mn/Fe ratio of Lago di Baratz indicating lake-level variability; (g) Accesa lake-level reconstruction after Magny et al. (2007a, b); (h) July temperature anomalies based on chironomids (Lago Verdarolo and Lago Gemini, Samartin et al. 2017) and (i) July and January insolation curves as anomalies to present-day values after Laskar et al. (2004)

Cross-correlation and RDA analyses show that the early dominance of *Erica* species at Lago di Baratz was closely connected to high charcoal-inferred fire activity (Figs. 7, 8). Lignotubers allow *E. arborea* and *E. scoparia* to quickly resprout and regenerate after intense fires (Ojeda et al. 2000; Keeley et al. 2012). Moreover, *Erica* species are highly combustible (Fernandes et al. 2000; Curt et al. 2011). Thus, increasing fire frequency may have favoured *Erica* populations (Teshome and Glatzel 2018). Although *Erica* species can be competitive under moist conditions, they are more drought-tolerant relative to other Mediterranean species such as *Q. ilex* (Lloret et al. 2004; Nogués et al. 2014; Parra and Moreno 2017). Eco-physiological findings from the island of Elba, northeast of Sardinia, and central Spain support this notion by providing ample evidence that *E. arborea* is extremely well drought-adapted (Battipaglia et al. 2014; Parra and Moreno 2017). This property explains why *Erica* species perform very well on sandy well-drained soils, with water stress, e.g. on dunes or sandstones (Stevenson 1985; Ojeda et al. 2000). Frequent drought spells and high fire activity have therefore been suggested to explain the early dominance of *Erica* at Sa Curcurica, an interpretation that is supported by the low lake-level stands at that time in Central Italy (Fig. 9, Beffa et al. 2016). A further common vegetation pattern between Lago di Baratz and Sa Curcurica is the early macrofossil-inferred dominance of *E. scoparia* and its gradual replacement by *E. arborea* during the Mid and Late Holocene. This similarity may mean that the shift between the two *Erica* species is characteristic for Holocene coastal ecosystems in Sardinia; however, more macrofossil evidence from other sites is needed to thoroughly address this issue.

It has been hypothesized that a Mid to Late Holocene trend to less seasonal climate, involving cooler and moister summers and warmer and drier winters, may have altered the vegetation composition and the fire regime, driving the gradual replacement of disturbance-adapted (fire, drought) *Erica* species with late successional *Q. ilex* on millennial timescales (Beffa et al. 2016). The long-term decrease of seasonality may have resulted from changes in orbital forcing from the Early to the Late Holocene (Berger and Loutre 1991; Kutzbach and Webb 1993; Fig. 9). The reduced seasonality hypothesis is consistent with reconstructions from Italy and other sites along the Mediterranean borderlands, suggesting that Holocene seasonality changes may have affected the hydrological regimes (Magny et al. 2007a, b, 2012; Roberts et al. 2010; Giraudi et al. 2011; Calò et al. 2012; Zanchetta et al. 2013; Curry et al. 2016; Wagner et al. 2019). Indeed, a gradual shift to less pronounced seasonality at ca. 7,500–5,500 cal bp (Curry et al. 2016; Budsky et al. 2019) may have involved diminishing erosion and/or floods, as reconstructed at Lago di Baratz. Moreover, decreased summer aridity and cooler summer conditions would have resulted in less fire activity. In agreement, recent chironomid-inferred temperature reconstructions show a marked Late Holocene summer temperature decline over Northern and Central Italy (Samartin et al. 2017) that most probably augmented Late Holocene summer moisture availability (Henne et al. 2013) after the Holocene Thermal Maximum (HTM). More specifically, it has been hypothesized that the centennial-scale climate cooling and resulting moisture increase at the end of the HTM (Samartin et al. 2017; Fischer et al. 2018) may have favoured the first mass expansion of *Q. ilex* at Sa Curcurica at around 5,500–5,000 cal bp. In agreement with the Sa Curcurica record (Beffa et al. 2016), the mass expansion of *Q. ilex* is dated to ca. 5,500–5,000 cal bp at Lago di Baratz, when deep lake levels suggest high moisture availability. In best agreement, the mass expansion of *Q. ilex* is dated at ca. 5,500 cal bp also on Cavallo, an island between Sardinia and Corsica, longitudinally located between Lago di Baratz and Sa Curcurica (Poher et al. 2017). A major difference between the records is, however, the role of *Pistacia* shrublands, which prominently replaced *Erica* woodlands at Lago di Baratz but not at Sa Curcurica and on Cavallo (Beffa et al. 2016; Poher et al. 2017).

Differences in geology and moisture availability may explain why *P. lentiscus* and *Q. ilex* were generally more important, and *Erica* species less important, at Lago di Baratz in western Sardinia than at Sa Curcurica in the east during the past 8,000 years (Fig. 9). In the Mediterranean region, vegetation varies according to bedrock, which in turn determines important soil properties such as pH and carbonate content (Brullo et al. 2008; Marcenò and Guarino 2015). Specifically, *P. lentiscus* and *Q. ilex* are competitive on soils deriving from calcareous bedrock, while *E. scoparia* and *E. arborea* are only competitive on siliceous, acidic soils (Chiappini 1985; Pignatti 2005). Jurassic limestone outcrops are relatively widespread east and south of Lago di Baratz, still within its pollen source area, while soils around Sa Curcurica, ca. 130 km east of Lago di Baratz, developed on Variscan granitoids and Plio-Pleistocene volcanic rocks (Cuccuru et al. 2015). Thus, the higher relevance of *Erica* in the east as well as the higher abundances of *Pistacia* in the west during the past 8,000 years can be explained by the local geology and the derived soil properties. The Cavallo record confirms this interpretation, given that rather low Holocene *Pistacia* abundances comparable to those at Sa Curcurica are associated with a geology that is characterized by granitic substrates (monzogranites and granodiorites; Poher et al. 2017). During the period 8,100–5,500 cal bp, the main competitor that replaced *Erica* at Lago di Baratz was *Pistacia* and to a lesser degree, *Q. ilex*, which only after 5,500 cal bp became more abundant in the west (peaks at ca. 30–35%) than in the east (peaks at 15–25%, Fig. 9). Again, the Cavallo record is in good agreement with the Sa Curcurica record in the east (peaks at ca. 15%; Poher et al. 2017). Mid Holocene environmental conditions involving high seasonality with dry and hot summers (Calò et al. 2012; Curry et al. 2016; Budsky et al. 2019) were likely more suitable for *P. lentiscus* than for *Q. ilex,* given that the latter species is less drought-tolerant (Pignatti 2005). Climatic comparisons (Table 1) show that due to temperature differences of ca. 1 °C and similar precipitation amounts, moisture availability at present is slightly higher in the west than in the east (Table 1). Probably of greater importance, in Sardinia rainfall is far more variable in the east than in the west (standard deviation of annual precipitation 250–500 vs 100–150 mm, SardegnaARPA 2020). Westerly winds bring more rainfall to the western parts of the island, however, while the topography is rather flat in the west, the presence of high mountain peaks in the east can cause heavy rainfall that may last for days in response to humid easterly winds (SardegnaClima 2021). Because westerly winds are frequent but easterly winds rare, rainfall variability is far higher in the east than in the west (SardegnaARPA 2020; SardegnaClima 2021). Since it is likely that this longitudinal moisture variability gradient also existed in the past, it may have contributed to different abundances of *Q. ilex*, *Pistacia* and *Erica*. Complex moisture/soil interactions may also explain why the dominance of *Erica* woodlands ended earlier at Lago di Baratz (ca. 5,500 cal bp) than at Sa Curcurica (ca. 2,800 cal bp, Fig. 9). However, the Late Holocene expansion of *Q. ilex* is also documented at other sites in Sardinia (Di Rita and Melis 2013; Melis et al. 2018), suggesting that this most prominent thermo-mediterranean vegetation change occurred across Sardinia.

In contrast to the pronounced Mid-Holocene flood, lake-level, fire and vegetation variability, environmental conditions were rather stable at Lago di Baratz during the Late Holocene. In agreement with previous multiproxy studies including dynamic vegetation modelling (e.g. Colombaroli et al. 2007; Henne et al. 2015; Beffa et al. 2016; Curry et al. 2016), no marked trend to drier Late Holocene conditions can be inferred from our new multiproxy record. The “aridification” signal observed in pollen diagrams (e.g. Jalut et al. 2009; Mercuri et al. 2012; Jiménez-Moreno et al. 2015; Ramos-Román et al. 2018; Schröder et al. 2018) could therefore primarily derive from land use, through the disruption of forests and the promotion of xerophilous fire- and grazing-adapted maquis communities (Colombaroli et al. 2007; Tinner et al. 2009, 2016; Bisculm et al. 2012). It may therefore be considered as the result of a human-driven xerophytization process that would cease in absence of excessive human impact (Henne et al. 2013, 2015).

### Human impact

First archaeological evidence of Neolithic farming belonging to the Italian Cardial culture is dated to 8,000–7,700 cal bp in Sardinia (Trump 1983; Ucchesu et al. 2017a). From 7,650–7,250 cal bp (5700 to 5300 cal bc), the Early Neolithic consolidation phase on the Tyrrhenian shores of Italy as well as in Corsica and Sardinia shows a synchronous archaeological evolution (Lugliè 2018; Revelles et al. 2019). Around 7,300 cal bp, most probably as a consequence of new influences from the European continent and specifically Northern Italy, the Neolithisation in Sardinia was accomplished and the material culture separated from Corsica and mainland Italy (Lugliè 2018). During the Neolithic, Sardinia became the most important area for obsidian tool production in the Mediterranean (Lugliè 2012; De Francesco et al. 2017). Archaeobotanical remains from crop production are rare in Sardinia for the Early Neolithic (sixth millennium bc). Pollen (e.g. Cerealia-t., *Plantago lanceolata*-t.) and macrofossil data suggest marginal agricultural and pastoral activities since ca. 8,000 cal bp (6050 cal bc), when sedimentation started at Lago di Baratz and *E. scoparia* and *E. arborea* were dominant. Although we assume that the *Erica* dominance was natural, archaeobotanical findings from the Neolithic period point to the use of *E. arborea* in fireplaces and as fodder for goats and sheep (Ucchesu et al. 2017a). The extremely high values of dung fungal spores (*Sporormiella*) during this early phase might be related to livestock farming, or perhaps more likely, to wild animals coming close to the lake to access freshwater, when the climate was probably dry and lake levels low.

After ca. 7,500 cal bp (5550 cal bc), land use became more intense in the Lago di Baratz area, as revealed by a first prominent Cerealia-t. peak around 7,250 cal bp (5300 cal bc). Similar evidence of early cereal cultivation comes from lowland lakes in Corsica (Revelles et al. 2019) and on Cavallo (Poher et al. 2017). In very good agreement with the Corsican evidence, a last major peak in the dung fungal spores *Sporormiella* occurred at 6,500–6,100 cal bp (ca. 4550–4150 cal bc) in the Lago di Baratz area, when indicators of pastoral activity (*Urtica*, *Rumex acetosa*-t., *R. acetosella* t., Poaceae) and arable farming (Cerealia-t.) slightly increased. The concomitant decline of *Q. ilex-*t. and *E. arborea*-t. may therefore be related to the need of new open land for pastures and fields. Our interpretation is in agreement with new bioarchaeological data from Sardinia, which document that land use was based on sheep, pigs, cows and cereals during the Middle Neolithic (Ucchesu et al. 2017a). Furthermore, the archaeobotanical evidence shows that Neolithic people also collected fruits of wild *Ficus carica*, *Olea europaea*, *Pinus* and *Pistacia lentiscus* (Ucchesu et al. 2017a). On Cavallo, expansion of *F. carica* trees occurred around 6,100 cal bp (Poher et al. 2017), whereas in Sicily figs were already cultivated with cereals at ca. 7,500 cal bp (Tinner et al. 2009). *Olea europaea* and *Pinus* produce edible fruits or seeds, while the fruits of *P. lentiscus* are useful for oil production, its wood is an excellent fuel for ceramic firing and its resin is used as a spice, adhesive, incense or medicine (Loi 2013; Sabato et al. 2015; Ucchesu et al. 2017a). Indeed, the pollen record suggests that *Pistacia* was abundant around Lago di Baratz, likely due to natural succession under the climatic conditions of that time, and provided valuable resources.

Our macrofossil and pollen records unambiguously document that during the Neolithic, *E. arborea* was widespread around Lago di Baratz. The wood of the species produces enduring embers, e.g. for cooking and heating, and the plant is used as fodder for goats and sheep (e.g. Atzei 2003; Ucchesu et al 2017a). Archaeobotanical and archaeological evidence points to the cultivation of *Triticum dicoccum*, *Hordeum vulgare* var. *nudum* and *Lens culinaris* close to our study site during the late Neolithic ca. 5,800 cal bp (ca. 3800 cal bc, Sa Ucca, Sassari, Bakels 2002; Ucchesu et al. 2017a). Generally, Late Neolithic or Copper Age agriculture during the fourth and third millennium cal bc was based on the cultivation of *Hordeum, Triticum* and Fabaceae as well as animal husbandry with sheep, goats, cows and pigs (Melis 2009; Ucchesu et al 2018). The presence of *Linum* remains in settlements during this time points to the cultivation of this fiber and oil plant, although nine species of wild flax grow today in Sardinia (Ucchesu et al. 2018). In best agreement, our pollen record shows the first appearance of *Linum* around 5,500 cal bp (ca. 3500 cal bc, Fig. 3b). After 5,500 cal bp (ca. 3500 cal bc), evergreen oak forests expanded at the expense of open habitats, *E. arborea* and *P. lentiscus* (Fig. 3a), to reach a maximum at around 3,700 cal bp (ca. 1750 cal bc, 40%), when fire incidence (charcoal record) and human impact (e.g. Cerealia-t., *Plantago lanceolata*-t.) was reduced to a minimum. This vegetation pattern suggests that the expansion of *Q. ilex* was not primarily a consequence of fire disturbance or land use activity, as also observed at other sites in Sardinia, Sicily, Italy and southern Croatia (e.g. Colombaroli et al. 2009; Tinner et al. 2009; Beffa et al. 2016). However, this evidence is in contrast with earlier hypotheses attributing the expansion of *Q. ilex* in Corsica to human impact (Reille 1992b) or increasing fire incidence (Carcaillet et al. 1997).

After ca. ca. 3,700 cal bp (1,750 cal bc), more specifically during the period 3,500–2,500 cal bp (ca. 1,550–550 cal bc), open land (NAP from ca. 20 to 40%) and maquis (e.g. *Pistacia*, *Cistus*, *Genista*-t.) expanded at the expense of *Q. ilex* (from ca. 30 to 10%). This forest decline was associated with the reorganization of aquatic ecosystems, as evidenced by the disappearance of RABD_837_-inferred meromixis. We associate this marked change in terrestrial and aquatic ecosystems with the establishment of the Nuraghi culture during the Bronze Age (Holt 2014). Our pollen record suggests that the Nuraghi culture in the Lago di Baratz area had specialized more in pastoral farming than in crop production. While cereal production remained comparable to that in the Neolithic, marked increases of e.g. *Rumex acetosa*-t., *Plantago lanceolata-*t., *Artemisia*, *Stachys*-t. and Poaceae point to substantial enlargement of pastures and fallow land. Farming also included plantations of fruit trees such as *Juglans regia* (first pollen found around 3,600 cal bp, ca. 1,650 cal bc). Archaeobotanical evidence confirms this pattern and shows that during the Bronze Age, Sardinian agriculture included the cultivation of cereals (*Hordeum* naked and hulled, *Triticum aestivum* and *T. durum*) and Fabaceae (*Vicia faba, Lens culinaris, Pisum sativum*; Ucchesu et al. 2014). Seeds and fruits of *Ficus carica* (5,000,000 achenes in 4 L)*, Vitis vinifera* (c. 15,400 pips in 0.8 L), *Olea europaea*, *Prunus spinosa*, *Rubus* sp., as well as *Pistacia*, show the local relevance of gathering or production of these edibles (Ucchesu et al. 2014, 2015; Sabato et al. 2015).

Today, *O. europaea* is the most important fruit tree around the Mediterranean Sea. The question of when *Olea* cultivation started remains open, as olives can also be harvested from wild plants (oleasters). Our data show that *O. europaea* was a natural component of local forest vegetation, associated with late-successional *Q. ilex* during the period 8,000–3,500 cal bp. For instance, the species expanded at around 5,500 and 3,700 cal bp (ca. 3550 and 1750 cal bc) when *Q. ilex* woodlands reached maximum abundances and land use (e.g. Cerealia-t., *R. acetosa*-t.) and fires (charcoal) were strongly reduced (Figs. 3, 4). This close link between *Q. ilex* and *O. europaea* has also been observed for the thermo-mediterranean belt of Sicily, unambiguously showing the ability of the tree to naturally compete in Mediterranean evergreen broadleaved forests (Tinner et al. 2009), a palaeoecological finding which is supported by current ecological evidence (Gianguzzi and Bazan 2019).

Around 3,300–3,100 cal bp (1,350–1,150 cal bc) this linkage between the two species is interrupted. Indeed, pollen data from Lago di Baratz point to an expansion of *Olea* when *Q. ilex* forests were drastically reduced. Chronologically, this decoupling corresponds to the period with first archaeobotanical evidence of wine and melon cultivation in Sardinia (Sabato et al. 2015; Ucchesu et al. 2015). No older melon cultivation is currently available from the Mediterranean (Sabato et al. 2015), suggesting that during the Bronze Age Sardinia was a center of agricultural innovation. Olive cultivation possibly started during the Copper Age (Chalcolithic culture) in the Levant, however, the fruits were traded in the Mediterranean realm at least since 3,300 bp cal (1350 bc), as illustrated by 2,500 olive remains found in a shipwreck in the Eastern Mediterranean (Kaniewski et al. 2012). On the basis of our palaeoecological record together with the archaeobotanical evidence of fruit cultivation, we assume that by 3,300 bp (1,350 cal bc), olive fruit production had started in Sardinia as suggested also for south-eastern Italy (Apulia) (Caracuta 2020). Intriguingly, the so-called Late Bronze Age collapse in the Near East, which shifted the economic centres towards Greece and Italy, was dated with olive remains to 3,140 cal bp (1190 cal bc, Kaniewski et al. 2012).

The alteration of vegetation through land use likely included the spread of *Q. suber* (cork oak). In our record, cork oak stands expanded and remained at a constant level over millennia from ca. 2,500 cal bp (550 cal bc) onwards. We assume that during the Phoenician and Punic periods humans fostered *Q. suber* for bark collection, whereas acorns may have served as fodder for domestic animals. Their use as human food is documented by the production of *Q. suber*, *Q. ilex* and *Q. pubescens* acorn bread (Pinna 2013). Among these three oak species *Q. suber* is considered to produce the sweetest acorns and thus the best bread (Atzei 2003). Similarly, in southern Italy acorns of evergreen and deciduous oak were also part of the human diet (Primavera and Fiorentino 2013). It is likely that the expansion of *Q. sub*er at 2,500 cal bp was the consequence of cork oak plantations and/or human facilitation. Indeed, the close linkage with cultural indicators (e.g. Cerealia-t., *Plantago lanceolata*-t. and *Urtica*), as well as to the minimum of *Q. ilex*, suggests a marked connection to agricultural practices. However, *Q. suber* declined after ca. 800 cal bp to slightly re-expand during the past 300 years. Archaeobotanical analyses underline the importance of fruits during the sixth to second centuries bc (Phoenician and Punic periods). For instance, cultivated plums (*Prunus domestica*), watermelon (*Citrullus lanatus*)*,* walnut (*Juglans regia*) and almonds (*Prunus dulcis*) were found in amphorae (Ucchesu et al. 2017b; Sabato et al. 2019)*.* The relevance of the fruit trees *Juglans regia* and *Prunus* is well documented in our pollen record at that time. Other planted fruit trees were *Citrus* after 1,200 cal bp and *Castanea sativa* during the past 500 years. Finally, during the past 300–200 years, cereal production intensified and pine plantations became ubiquitous, mainly at the expense of *Q. ilex* forests in the Lago di Baratz area. Exotic trees such as *Eucalyptus* were also introduced during the past decades, further reducing the area of native Mediterranean woody communities. This last anthropogenic change of vegetation cover had unexpected consequences, given that the potential benefits such as the increase of timber production were counteracted by an increase in flammability and thus fire risk. Indeed, because of their resins and oils, *Pinus* and *Eucalyptus* are among the most flammable trees planted in the Mediterranean, while native thermo-mediterranean *Q. ilex*, *Q. suber* and *O. europaea* are less susceptible to combustion (Fernandes 2009; Moreira et al. 2009).

## Conclusions

Our study shows that during the Mid and Late Holocene, *Erica* woodlands were gradually replaced by evergreen mixed oak forests. The expansion of mixed *Q. ilex* forests with a considerable share of *O. europaea* was associated with a decline of fire activity, as also observed at many other sites in the Central Mediterranean (e.g. Colombaroli et al. 2009; Tinner et al. 2009; Beffa et al. 2016). We assume that this paramount ecosystem change was mainly the consequence of changing climate, which promoted less continental (warmer winters, cooler summers) conditions that gradually reduced drought disturbance. Erosion declined and aquatic ecosystems stabilized in association with Mid Holocene climate change and the resulting fire decrease and evergreen mixed oak forest expansion. Bedrock and soil differences influenced the Holocene vegetation composition in Sardinia, as previously reconstructed in other areas (e.g. Northern Italy, Gobet et al. 2000). Excellent bioarchaeological records (e.g. Ucchesu et al. 2014, 2015; Sabato et al. 2015) allow detailed insights into prehistoric land-use practices and corroborate our interpretation that human impact became pervasive since the Bronze Age, overrunning climate as the main determinant of ecosystem change during the past 4,000 years. Human impact during the Nuraghi period resulted in the disruption of evergreen broadleaved forests for cultivation of crops such as cereals, olive, grapes and melons. Subsequently, human disturbance increased further and ultimately led the mass expansion of grasslands, maquis and garrigues, resulting in an artificial xerophytization process, which apparently mimicked an aridification signal. Taken together, the available evidence allows us to conclude that in the absence of human impact, the warmest and driest coastal environments of Sardinia would still be covered by mixed *Q. ilex*-*O. europaea* woodlands, a finding which is in agreement with modern ecological inferences (e.g. Chiappini 1985; Bacchetta et al. 2003; Gianguzzi and Bazan 2019). This result is of great importance to reduce future hazards, such as increasing fire and flood impacts. In fact, our data suggest that re-establishing natural mixed *Q. ilex* woodlands may help reduce soil erosion, e.g. in response to floods. Furthermore, a replacement of flammable maquis, garrigue, pine and eucalypt plantations (Fernandes 2009; Moreira et al. 2009) by native *Q. ilex* and *O. europaea* woodlands may significantly reduce fire hazards for forests and infrastructure, aiding in maintaining sustainable ecosystem services (Henne et al. 2015).
